# A poroelastic mixture model of mechanobiological processes in biomass growth: theory and application to tissue engineering

**DOI:** 10.1007/s11012-017-0638-9

**Published:** 2017-02-20

**Authors:** Riccardo Sacco, Paola Causin, Chiara Lelli, Manuela T. Raimondi

**Affiliations:** 1 Dipartimento di Matematica, Politecnico di Milano, Piazza Leonardo da Vinci 32, 20133 Milano, Italy; 2 Dipartimento di Matematica “F. Enriques”, Università degli Studi di Milano, Via Saldini 50, 20133 Milan, Italy; 3 Present Address: Via IV Novembre, 80, 51030 Marliana (PT), Italy; 4 Dipartimento di Chimica, Materiali e Ingegneria Chimica “Giulio Natta”, Politecnico di Milano, Piazza Leonardo da Vinci 32, 20133 Milan, Italy

**Keywords:** Tissue engineering, Mechanobiology, Mathematical modeling, Mixture growth theory, Mass transport, Continuum mechanics, Numerical simulation

## Abstract

In this article we propose a novel mathematical description of biomass growth that combines poroelastic theory of mixtures and cellular population models. The formulation, potentially applicable to general mechanobiological processes, is here used to study the engineered cultivation in bioreactors of articular chondrocytes, a process of Regenerative Medicine characterized by a complex interaction among spatial scales (from nanometers to centimeters), temporal scales (from seconds to weeks) and biophysical phenomena (fluid-controlled nutrient transport, delivery and consumption; mechanical deformation of a multiphase porous medium). The principal contribution of this research is the inclusion of the concept of cellular “force isotropy” as one of the main factors influencing cellular activity. In this description, the induced cytoskeletal tensional states trigger signalling transduction cascades regulating functional cell behavior. This mechanims is modeled by a parameter which estimates the influence of local force isotropy by the norm of the deviatoric part of the total stress tensor. According to the value of the estimator, isotropic mechanical conditions are assumed to be the promoting factor of extracellular matrix production whereas anisotropic conditions are assumed to promote cell proliferation. The resulting mathematical formulation is a coupled system of nonlinear partial differential equations comprising: conservation laws for mass and linear momentum of the growing biomass; advection–diffusion–reaction laws for nutrient (oxygen) transport, delivery and consumption; and kinetic laws for cellular population dynamics. To develop a reliable computational tool for the simulation of the engineered tissue growth process the nonlinear differential problem is numerically solved by: (1) temporal semidiscretization; (2) linearization via a fixed-point map; and (3) finite element spatial approximation. The biophysical accuracy of the mechanobiological model is assessed in the analysis of a simplified 1D geometrical setting. Simulation results show that: (1) isotropic/anisotropic conditions are strongly influenced by both maximum cell specific growth rate and mechanical boundary conditions enforced at the interface between the biomass construct and the interstitial fluid; (2) experimentally measured features of cultivated articular chondrocytes, such as the early proliferation phase and the delayed extracellular matrix production, are well described by the computed spatial and temporal evolutions of cellular populations.

## Introduction

**Fig. 1:**
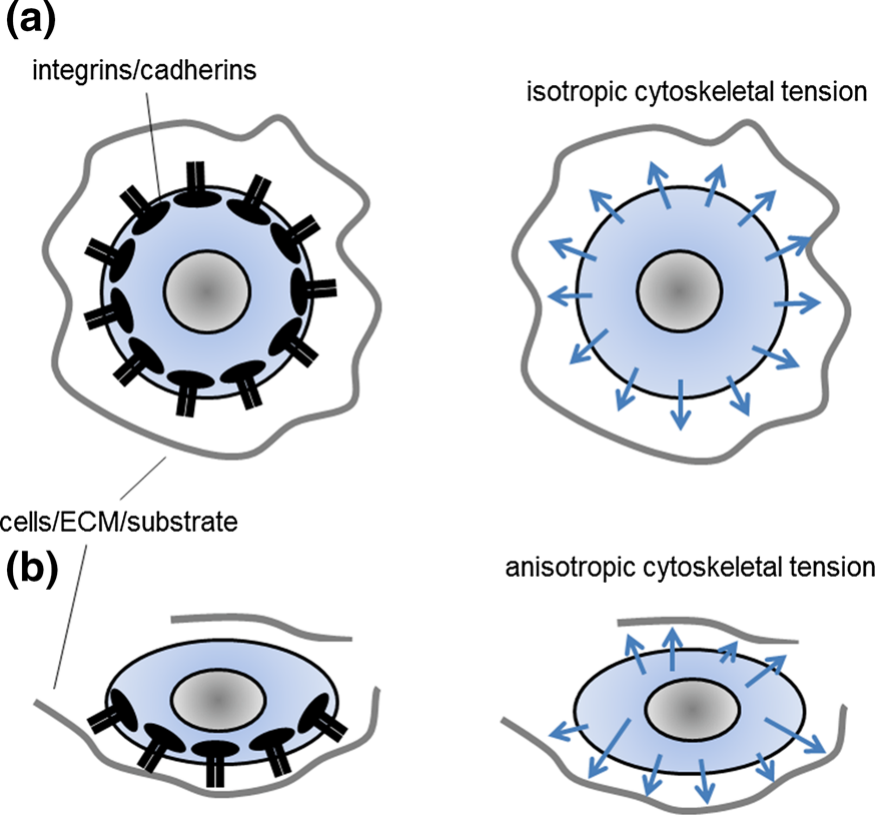
Concept of isotropic/anisotropic stress state of a cell: **a** a uniform distribution of traction forces (mediated by cell membrane integrin/cadherins) over the cell surface generates an “isotropic cytoskeletal tension state” in which the cell nucleus tends to maintain a roundish morphology, favoring ECM secretion; **b** an “anisotropic cytoskeletal tension state” the cell nucleus tends to elongate, favoring cell mitosis

It is nowadays a founding concept in cellular and molecular biology that cells are able to sense mechanical stimuli in their surrounding environment and produce a coordinate response. Such a process, defined as *mechanotransduction* (see, e.g., the recent review in [50]), plays important roles in several physiological processes such as cell motility, angiogenesis, bone formation and wound healing [76]. In this work, we present a mathematical approach for describing mechanotransduction processes involved in tissue growth. The proposed description, albeit very general, is applied to the scenario represented by tissue engineering. In this context, a better knowledge of the role of biomechanical cues can help in orchestrating a more effective artificial tissue growth. More in detail, our work is motivated by a specific tissue engineering application, artificial regeneration of articular cartilage. Briefly, cartilage cells (articular chondrocyte cells, ACCs) or other progenitor cells are seeded into polymeric scaffolds, possibly perfused by an interstitial fluid to force nutrient delivery. Cells are expected to duplicate and, above all, produce an increasing mass of ECM, forming cartilagineous neo-tissue. Cartilage tissue growth in engineered constructs had been already studied in a series of papers by Klisch and coauthors [30–32]. In this work, we enrich the description of the biophysical phenomena by introducing the conceptual framework developed in [43, 45, 46]. In these works, the isotropic/anistropic state of the cytoskeletal tension is shown to be responsible for triggering signalling transduction cascades which regulate functional cell behaviors related to proliferation and/or ECM secretion. Under the assumption of an isotropic strain-stress response, a uniform distribution of stress over the cell surface - stress due to the traction forces exerted by the cell on the surrounding environment - generates an “isotropic cytoskeletal tension state” in which the cell nucleus tends to maintain a roundish morphology (see Fig. 1a). Conversely, in an “anisotropic cytoskeletal tension state” the cell nucleus tends to elongate (see Fig. 1b). Macroscopically speaking, when the nucleus maintains a roundish morphology, ECM secretion is favoured, whereas an elongated nucleus favors cell duplication by dividision along a polarization axis represented by its longer axis itself. According to a finer biomolecular view, this process can be interpreted as due to the fact that the shape of the nucleus is known to regulate the porosity of its membrane and, through this, the import flow of specific transcription factors which regulate cell behavior (we refer to [43] and references therein for a more biologically detailed discussion of these complex processes).

**Fig. 2:**
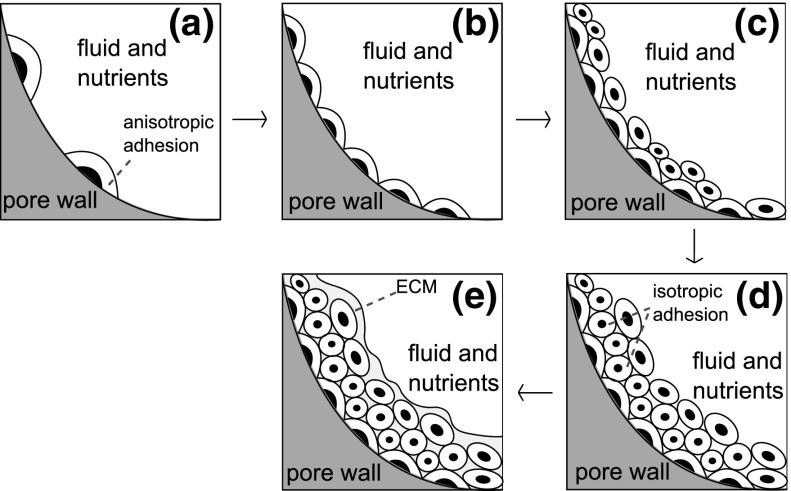
Various phases of tissue growth inside a scaffold pore: **a** seeding phase and cell polarization; **b** proliferation and formation of a monolayer; **c**, **d** formation of new construct layers; **e** ECM secretion

In order to work in a continuum mechanics framework, we propose an extension of the above concepts to aggregates of cells. Figure 2 schematically illustrates how the mechanobiological conditions may affect and drive the fate of a colony of ACCs seeded in a 3D porous scaffold. When cells are first seeded in the scaffold, they form a thin layer covering the surface. Since the characteristic dimension of the local scaffold curvature is much larger than cell size, cells find themselves in a local planar condition (see Fig. 2a). Exerting adhesion forces on the scaffold surface, cells tend to assume a spread elongated shape, orienting themselves along a preferred polarization axis. According to the concept of “force isotropy”, this represents a condition which enhances the probability that the single cell enters into a proliferative state. This situation persists until all the pore surface is covered with cells (Fig. 2b). From this moment on, cells start to occupy the empty space of the pore (Fig. 2c, d). Cells in contact with other cells sense an isotropic stress state condition, which drives the cell towards a mature differentiated phenotype, characterized by ECM secretion (Fig. 2e).

To translate the mechanobiological description of the processes illustrated in Fig. 2 into a mathematical model, we combine the poroelastic theory of mixtures and cellular population models. Using the model, we predict the spatial and temporal distribution of a biomass aggregate of ACCs and ECM under the biophysical assumption that stress state and oxygen (nutrient) tension act as main determinants of engineered culture evolution. The poroelastic theory of mixtures has already been proposed elsewhere to describe mechanobiological processes in growing tissues, possibly combined with a multiscale approach. In these formulations, tissue growth is represented as mass exchange between phases in a globally mass-conserving framework. Moreover, the assumption of linear strains is made, justified by the fact that the microscopic representative volume in which the volume averaging is performed does not evolve in time whereas the individual phases do. We refer for these approaches to the comprehensive discussion in [5], where several different techniques (effective medium theory, mixture theory, volume averaging and asymptotic (two-scale) homogenization) available to describe a poroelastic growing system are discussed and compared. Moreover, we refer to [54] for an example of the use of asymptotic homogenization techniques to develop a model for growing poroelastic media. As for cellular population models, they are used in several literature papers to describe the evolution of mixture components. We refer in particular to the works of [51] and [67], where multiple cellular populations are studied describing the exchange from one population to the other via a phenomenological representation. From this perspective, the principal contribution of our mechanobiological model is the inclusion of the concept of cellular “force isotropy” as a determinant of the passage from one pool of cells to the other (proliferating, ECM secreting o quiescent cells). A phenomenological indicator of the stress/strain state of the continuum construct is proposed, based on the on the norm of the deviatoric part of the total stress tensor computed via the poroelastic theory. Similarly to other models in tissue engineering applications (see, e.g., [14, 47], we also include the effect of nutrient (oxygen) availability by solving for it a transport-diffusion-reaction equations. Nutrient levels are supposed to be as well driving mechanisms in the cellular pool exchange. We use the model on a preliminary simplified geometrical one-dimensional setting to study the influence of the different parameters on the evolution of the construct composition. The extensive numerical simulations carried out under different working conditions show that fundamental roles are played by the maximum cell specific growth rate and by the mechanical boundary conditions at the interface between biomass construct and interstitial fluid.

The paper is organized as follows: in Sect. 2, we present the assumptions leading to the description of the biomass as a mixture; in Sect. 3, we discuss the kinematic laws for the growing biomass; in Sect. 4 we formalize the balance laws for the biomass; in Sects. 5 and 6 we discuss the proposed exchange pathways interconnecting biomechanical cues and cell population evolution along with the corresponding model; in Sect. 7, we present the 3D mathematical model with the boundary conditions, while in Sect. 8 we introduce the reduced 1D setting with the proposed stress indicator; in Sect. 9, we discuss the numerical approximation of the 1D model and in Sects. 10 and 11 we present the results of the numerical simulations and we carry out a comprehensive discussion. Eventually, in Sect. 12 we draw the conclusions and we present perspectives for future work.

## Multiphase modeling

In this section we develop a mathematical model based on the representation of the ensemble of the growing cartilaginous biomass by the mixture theory. In this framework, equations are postulated for the balance of mass and momentum for each constituent and then for the entire mixture according to the following ideas:

- the growing biomass is treated as a mixture composed by a multiphase solid mass and an interstitial fluid, the latter representing a fraction of the order of 65–80% in mass of the total biomass. The multiphase solid consists of ACCs and of ECM. ACCs are pooled in different populations according to their life cycle status (proliferative, ECM secreting, quiescent), as in the works of Sengers [66] and Ducrot [25];
- the poroelasticity theory is used to model the interaction of deformation and fluid flow in the fluid-saturated porous, elastic solid [6, 22];
- the kinematics of the solid phase of the mixture is based on an infinitesimal–deformation approach, including the effect on the stress field of biological growth, according to the formulation proposed by Klisch and co–authors [31–33];
- the mass conservation balance for each single constituent and for the mixture are written according to the formulation introduced by Lemon and co-authors [35, 36] and extensively analyzed in [51, 70];
- the mass exchange terms, including the rate of switch of cells from a population to the other, are tuned according to the nutrient level, the latter being itself an unknown of the problem, and to the stress state locally experienced by the mixture, which may drive cells into a certain functional behavior pool.

In the following, we use the term “phase” when we refer to the solid or to the fluid part of the mixture, while the term “component” is used to refer to any of the constituents of the solid phase (cell populations and ECM). When it is not necessary to distinguish between phase and component, we simply use the term “species”. The meaning of the subscripts used throughout the article is as follows: s=solid phase, fl=fluid phase, cells=cell component of the solid phase, ECM= extracellular component of the solid phase.

We let ${\mathbf {x}}$ and *t* denote the space and time variables, respectively. We use the convention that the dependence of all variables and model parameters on ${\mathbf {x}}$ and *t* is left understood except otherwise stated.

The geometrical configuration of the mixture is identified by the open bounded set $\varOmega \subset {\mathbb {R}}^d$ ($d=3$ unless otherwise specified). The domain $\varOmega$ does not evolve in time, rather, it is the amount of each species at a point ${\mathbf {x}} \in \varOmega$ that changes with *t* due to cell proliferation and matrix deposition. This is the precise sense of the concept of “growing mixture”. From now on, we denote by ${\mathcal {Q}}_{T_{end}}: = \varOmega \times (0, T_{end})$ the space-time cylinder in which the TE problem is studied, $T_{end} >0$ being the final time of culture process.

**Fig. 3:**
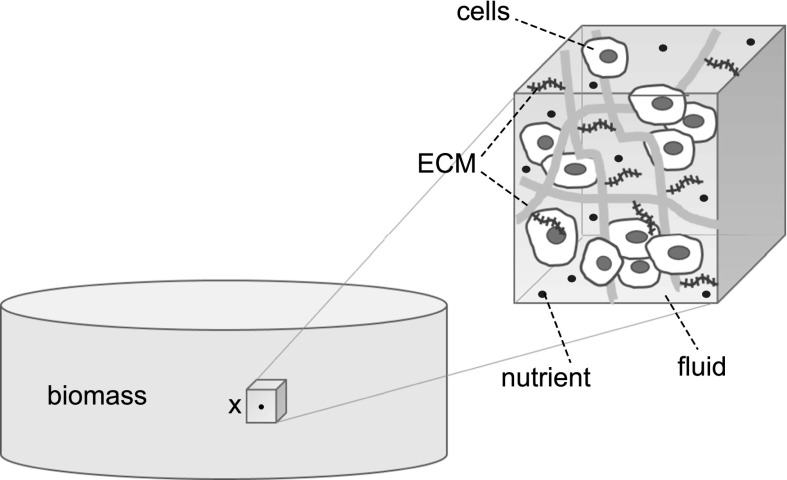
A schematic view of the computational domain $\varOmega$ with a detailed view of a typical REV where the various phases and components of the growing mixture are identified

Referring to Fig. 3, for all $t>0$ we associate with a generic point ${\mathbf {x}} \in \varOmega$ a fixed representative elementary volume (REV) ${\mathcal {V}}^{\mathbf {x}}$ (see [73]) and denote by $|{\mathcal {V}}^{\mathbf {x}}|$ its *d*-dimensional volume. Then, for each component $i={\text {fl}}, {\text {s}}$ of the growing mixture, we define the volume fraction

$$\begin{aligned} \phi _i ({\mathbf {x}},t) = \displaystyle \frac{|{\mathcal {V}}^{{\mathbf {x}}}_{i}({\mathbf {x}},t)|}{|{\mathcal {V}}^{\mathbf {x}}|} \qquad \forall {\mathbf {x}} \in \varOmega , \qquad \forall t >0 \end{aligned}$$

as the time evolving ratio of the volume occupied by the *i*-th component in the REV and the volume of the REV itself. We also let

1a $$\begin{aligned} \phi _{{\text {s}}}({\mathbf {x}},t) &=\phi _{{\text {cells}}}({\mathbf {x}},t) + \phi _{{\text {ECM}}}({\mathbf {x}},t) \\&\forall {\mathbf {x}} \in \varOmega , \qquad \forall t >0. \end{aligned}$$

According to the biochemical hypothesis a), we consider three ACC populations: proliferating, ECM secreting state and quiescent state, denoted by the letters ${\text {n}}$, ${\text {v}}$ and ${\text {q}}$, respectively. Then, we have

1b $$\begin{aligned} \phi _{{\text {cells}}}({\mathbf {x}},t) &=\phi _{{\text {n}}}({\mathbf {x}},t) + \phi _{{\text {v}}}({\mathbf {x}},t) + \phi _{{\text {q}}}({\mathbf {x}},t) \\&\forall {\mathbf {x}} \in \varOmega , \qquad \forall t >0. \end{aligned}$$

Eventually, we denote by ${\varvec{\phi }} = \left[ \phi _{{\text {fl}}}, \, \phi _{\text {n}}, \, \phi _{\text {v}}, \, \phi _{\text {q}}, \, \phi _{{\text {ECM}}} \right] ^T$ the vector-valued function comprising the volume fractions of the fluid phase, the three cellular populations and the ECM.

The following assumptions on the mixture are also considered.

### **Assumption 1**

(*Fully saturated mixture*) The mixture is fully saturated, i.e.

1c $$\begin{aligned}\phi _{{\mathrm {s}}}({\mathbf {x}},t) + \phi _{{\mathrm {fl}}}({\mathbf {x}},t) = 1\quad \forall {\mathbf {x}} \in \varOmega , \qquad \forall t >0. \end{aligned}$$

Relation (1c) is referred to as *saturation condition* [3, 51, 65] and excludes the possibility of the formation of voids or air bubbles inside the growing mixture.

### **Assumption 2**

(*Intrinsic incompressibility*) All species constituting the growing mixture have the same (constant) mass density $\rho _w$ of the physiological interstitial fluid assimilated to water [3, 26, 30, 36, 51]. This is not, in general, equivalent to assuming that the *whole* mixture is incompressible (see [51] p. 629).

### **Assumption 3**

(*Closed mixture*) The mixture is closed, this meaning that the system does not exchange mass with external mass sources or sinks [51].

## Kinematics of the growing mixture

From now on, we denote “solid matrix” the collection of solid phase constituents, that is, cells and ECM. Then, we apply to the solid matrix the so–called *intermingled mixture constraint* [2], stating that all the solid matrix constituents experience the same overall motion. This hypothesis amounts to assuming the displacement and velocity vectors of each constituent to coincide with those of the solid matrix. Then, we denote by ${\mathbf {u}}_{{\text {s}}}={\mathbf {u}}_{{\text {s}}}({\mathbf {x}},t)$ and ${\mathbf {v}}_{{\text {s}}}={\mathbf {v}}_{{\text {s}}}({\mathbf {x}},t)= \dfrac{\partial }{\partial t}{\mathbf {u}}_{{\text {s}}}({\mathbf {x}},t)$ the displacement and velocity at the time level *t* of any point ${\mathbf {x}}$ of the solid component of the biomass, and by ${\varvec{\varepsilon }}_{{\text {s}}}({\mathbf {x}},t) = \frac{1}{2} (\nabla {\mathbf {u}}_{{\text {s}}}({\mathbf {x}},t) + (\nabla {\mathbf {u}}_{{\text {s}}}({\mathbf {x}},t))^T)$ the associated infinitesimal deformation of the biomass volume surrounding the point ${\mathbf {x}}$ at time *t*. The intermingled mixture constraint yields also the following relation

2a $$\begin{aligned} {\varvec{\varepsilon }}_{\eta }= {\varvec{\varepsilon }}_{{\text {s}}} \qquad \eta ={\text {cells}},{\text {ECM}}. \end{aligned}$$

The growth process of each mixture component (cellular growth and ECM secretion) is taken into account by introducing the following decomposition [33]

2b $$\begin{aligned} {\varvec{\varepsilon }}_{\eta }={\varvec{\varepsilon }}_{\eta }^g+ {\varvec{\varepsilon }}_{\eta }^e, \qquad \eta ={\text {cells}},{\text {ECM}}, \end{aligned}$$

where ${\varvec{\varepsilon }}^g_{\eta }$ is the infinitesimal growth tensor associated with each solid constituent of the biomass, accounting for the amount and the spatial orientation of the newly deposited mass, and ${\varvec{\varepsilon }}^e_{\eta }$ is the elastic accommodation tensor necessary to reinforce at each time level the continuity of the whole solid upon growth. Finally, we denote by

2c $$\begin{aligned} {\mathbf {w}}={\mathbf {v}}_{{\text {fl}}}-{\mathbf {v}}_{{\text {s}}} \end{aligned}$$

the relative velocity [51, 65] of the fluid phase with respect to the solid phase in the biomass, ${\mathbf {v}}_{{\text {fl}}}$ being the velocity of the interstitial fluid. For notational brevity, from now on, we simply write ${\mathbf {u}}$ and ${\varvec{\varepsilon }}$ instead of ${\mathbf {u}}_{{\text {s}}}$ and ${\varvec{\varepsilon }}_{{\text {s}}}$, respectively.

## Balance laws for the deformable growing biomass

In this section we illustrate the set of conservation laws that model the mechanobiological processes regulating biomass tissue growth. For further discussion and analysis of mixture theory applied to tissue growth modeling we refer the reader to [11, 36, 51].

### Mass balance for mixture components

The mass balance equation for the growing mixture is given by the following coupled system of PDEs in conservation form to be solved in ${\mathcal {Q}}_{T_{end}}$:

3a $$\begin{aligned} \frac{\partial {\varvec{\phi }}}{\partial t}+ \mathbf{div }\,{\mathbf {J}}_{{\varvec{\phi }}} &={\mathbf {Q}}({\varvec{\phi }},c,{\mathbf {T}}) \end{aligned}$$

3b $$\begin{aligned} {\mathbf {J}}_{{\varvec{\phi }}} &=\left[ \phi _{{\text {fl}}} {\mathbf {v}}_{{\text {fl}}},\phi _{\text {n}}{\mathbf {v}}_{{\text {s}}},\phi _{\text {v}}{\mathbf {v}}_{{\text {s}}},\phi _{\text {q}}{\mathbf {v}}_{{\text {s}}},\phi _{{\text {ECM}}} {\mathbf {v}}_{{\text {s}}}\right] ^T \end{aligned}$$

3c $$\begin{aligned} {\mathbf {Q}} &=\left[ Q_{{\text {fl}}}, Q_{{\text {n}}}, Q_{\text {v}}, Q_{\text {q}}, Q_{{\text {ECM}}}\right] ^T&\end{aligned}$$

where ${\mathbf {J}}_{{\varvec{\phi }}} \in {\mathbb {R}}^{5 \times d}$ is the flux matrix and ${\mathbf {Q}}$ is the net mass production rate for which the following constraint holds, due to Assumption 3

3d $$\begin{aligned} \sum _{\zeta ={\text {s}},{\text {fl}}}Q_\zeta =0. \end{aligned}$$

Equation (3b) represents a phenomenological description of the flux density of each species under the effect of convective transport due to the fluid and solid velocity, respectively. It is worth noting that in cartilage tissue growth, cells do not typically exhibit a significant diffusive motion, rather, they need a solid support for surviving and for developing their functional activities (property of anchorage-dependence [47]). For this reason in the present work we neglect the contribution to the flux density due the the diffusive transport, unlike in other applications where this term plays a significant role [37, 41, 49].

### Momentum balance for mixture components

Under the assumption of negligible inertial terms and absence of body forces and volumetric fluid mass sources and sinks, the linear momentum balance equation for the solid and fluid phases of the growing mixture is expressed by the following PDEs in conservation form to be solved in ${\mathcal {Q}}_{T_{end}}$:

4a $$\begin{aligned}&\mathbf{div }{}{\mathbf {T}}_{\zeta } ({\mathbf {u}}, \, p, \, {\varvec{\phi }}) + {\varvec{\pi }}_\zeta = {\mathbf {0}} \qquad \zeta = {\text {s}}, {\text {fl}} \end{aligned}$$

4b $$\begin{aligned}&{\mathbf {T}}_{{\text {s}}}({\mathbf {u}}, \, p, \, {\varvec{\phi }}) = \sum _{\eta ={\text {cells}},{\text {ECM}}}\phi _{\eta } {\mathbf {T}}_{\eta }({\mathbf {u}}, \, p, \, {\varvec{\phi }}) \end{aligned}$$

4c $$\begin{aligned}&{\mathbf {T}}_{\eta }({\mathbf {u}}, \,p, \, {\varvec{\phi }}) = {\varvec{\sigma }}_{\eta }({\mathbf {u}}, \, {\varvec{\phi }}) - p {\mathbf {I}} \qquad \eta ={\text {cells}},{\text {ECM}} \end{aligned}$$

4d $$\begin{aligned}&{\mathbf {T}}_{{\text {fl}}}({\mathbf {u}}, \, p, \, {\varvec{\phi }}) = - \phi _{{\text {fl}}} \, p {\mathbf {I}}, \end{aligned}$$

where ${\varvec{\sigma }}_\eta$ is the effective stress tensor of the component $\eta$ of the solid phase of the mixture, $p=p({\mathbf {x}},t)$ is the pressure exerted by the fluid phase and ${\mathbf {I}}$ is the identity tensor. The isotropic stress $-p {\mathbf {I}}$ accounts for the coupling, typical of poroelasticity, between the flow of the fluid and the deformation of the solid matrix, and in particular describes the contribution to the stress due to the fluid pressure within the structure.

The quantities ${\mathbf {T}}_{\zeta }$, $\zeta = {\text {s}}, {\text {fl}}$, are the total stress tensors of the solid and fluid phases, while ${\varvec{\pi }}_\zeta$ are the interphase forces [36]. As usual, we neglect the effective stress tensor of the fluid, meaning that we assume that the internal fluid viscosity is negligible compared with the friction between the fluid and the solid matrix [4, 26, 51]. For the mathematical characterization of the forces ${\varvec{\pi }}_{\zeta }$ we refer to [36] and [51]. We observe that, for all $t \in (0, T)$ and at all ${\mathbf {x}}\in \varOmega$, it holds

4e $$\begin{aligned}&{\varvec{\pi }}_{{\text {s}}}({\mathbf {x}},t) + {\varvec{\pi }}_{{\text {fl}}}({\mathbf {x}},t) = {\mathbf {0}}.&\end{aligned}$$

### Total mass and momentum balance for the growing biomass

The mass balance equation for the whole growing mixture is obtained by summing each component in system (3a), using (2c) and Assumptions 1 and 3:

5a $$\begin{aligned}&{\mathrm {div}}\,{\mathbf {v}} = 0, \end{aligned}$$

5b $$\begin{aligned}&{\mathbf {v}}=\phi _{{\text {fl}}} {\mathbf {v}}_{{\text {fl}}} + \phi _{{\text {s}}} {\mathbf {v}}_{{\text {s}}}&\end{aligned}$$

where ${\mathbf {v}}$ is the composite velocity of the mixture (cf. Eq. (2.4) of [51]). A simple manipulation allows us to write Eq. (5b) as

5c $$\begin{aligned}&\displaystyle \frac{\partial }{\partial t}{\text {div}}{} {\mathbf {u}} + {\mathrm {div}}(\phi _{{\text {fl}}} {\mathbf {w}}) = 0.&\end{aligned}$$

In a similar manner, summing Eqs. (4) and using (4e), we get the total momentum equation

5d $$\begin{aligned}&{\mathbf {div}}{}{\mathbf {T}}({\mathbf {u}}, \, p, \, {\varvec{\phi }}) = {\mathbf {0}}&\end{aligned}$$

5e $$\begin{aligned}&{\mathbf {T}}({\mathbf {u}}, p, \, {\varvec{\phi }}) = \sum _{\eta = {\text {cells}}, {\text {ECM}}} \phi _{\eta }{\varvec{\sigma }}_{\eta } ({\mathbf {u}}, \, {\varvec{\phi }})- p {\mathbf {I}}.&\end{aligned}$$

where ${\mathbf {T}}$ is the total stress in the mixture.

### Mass balance for nutrient concentration

The mass balance system (3) for the solid and fluid phases of the growing mixture is accompanied by a corresponding continuity equation for the nutrient concentration (oxygen) $c=c({\mathbf {x}},t)$ that is transported throughout the growing on mixture by the interstitial fluid. This continuity equation is expressed by the following PDE in conservation form to be solved in ${\mathcal {Q}}_{T_{end}}$:

6a $$\begin{aligned} \frac{\partial c}{\partial t}+ {\text {div}}\,{\mathbf {J}}_{\mathrm {c}} &= Q_{\mathrm {c}}({\varvec{\phi }},c) \end{aligned}$$

6b $$\begin{aligned} {\mathbf {J}}_{\mathrm {c}} &={\mathbf {v}}_{{\text {fl}}} c -D_{\mathrm {c}} \nabla c, \end{aligned}$$

the interstitial fluid velocity ${\mathbf {v}}_{{\text {fl}}}$ being computed using (2c) as

6c $$\begin{aligned}&{\mathbf {v}}_{{\text {fl}}} = {\mathbf {w}} + {\mathbf {v}}_{{\text {s}}} = {\mathbf {w}} + \dfrac{\partial {\mathbf {u}}}{\partial t}.&\end{aligned}$$

The mathematical description of the oxygen diffusion coefficient $D_{\mathrm {c}}$ adopted in this article is the so-called Maxwell model [74], that allows to account, in a volume-averaged sense, for the microscopic composition of the biomass. More precisely, we introduce the effective diffusion coefficient

$$\begin{aligned} D_{\mathrm {c}}:=D_{{\mathrm {c}},{\text {fl}}}\dfrac{3k-2\phi _{\text {fl}}(k-1)}{3+\phi _{\text {fl}}(k-1)}, \qquad k:=K_{\mathrm {eq}}\dfrac{D_{{\mathrm {c}},{\text {s}}}}{D_{{\mathrm {c}},{\text {fl}}}} \end{aligned}$$

where $D_{{\mathrm {c}},{\text {fl}}}$ and $D_{{\mathrm {c}},{\text {s}}}$ represent the nutrient diffusivity in the fluid and solid phase, respectively, while $K_{\mathrm {eq}}$ is the coefficient regulating local mass equilibrium between nutrient concentration in the solid and fluid phases (see [74] for a detailed discussion).

The time rate of oxygen consumed by the cellular populations is modeled by a generalized form of the Michaelis-Menten kinetics

6d $$\begin{aligned}&Q_c({\varvec{\phi }},c) = - (R_{\text {n}}\phi _{\text {n}}+ R_{\text {v}}\phi _{\text {v}}+ R_{\text {q}}\phi _{\text {q}}) \displaystyle \frac{c}{c+K_{1/2}}&\end{aligned}$$

where $R_\eta$, $\eta ={\text {n}},{\text {v}},{\text {q}}$, is the nutrient consumption rate of the cellular population $\phi _\eta$ and $K_{1/2}$ is the half saturation constant. We refer to [63] and the literature cited therein for a similar treatment of the oxygen consumption term in the framework of a multi-phase growing mixture.

## Mass exchange pathways

The production terms $Q_{\eta }$, $\eta =$cells,ECM, introduced in Eq. (3c) mathematically describe the mechanisms of addition and/or removal of mass for each species constituting the biomass growing mixture.

**Fig. 4:**
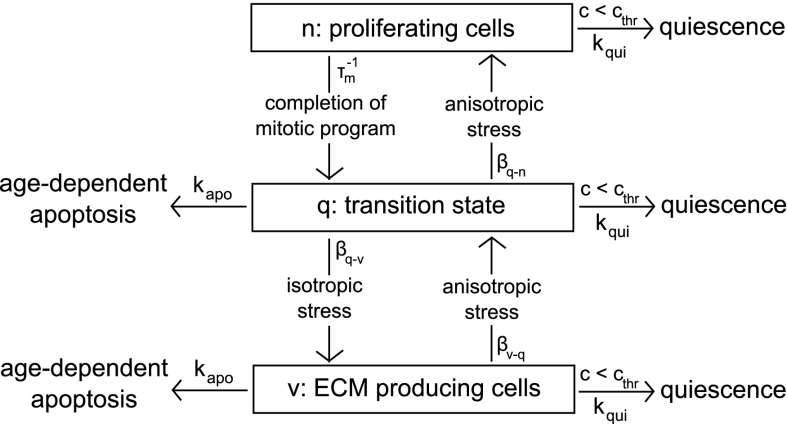
Conceptual scheme of exchange pathways among cellular populations (generalization of Fig. 5.3 in [67])

The exchange between the different functional cellular pools are supposed to be mediated by local population concentration, local stress state, local nutrient concentration and by natural decay times (see Fig. 4). To quantify the stress-mediated effect we proceed as follows. Let *H*(*z*) be the Heaviside function such that $H(z) =0$ for $z <0$ and $H(z) = 1$ for $z=0$, *z* being a real number. Then, the stress-state dependent effect is represented by $H(r-\overline{r})$, *r* and $\overline{r}$ being an indicator of the isotropy or anisotropy of the local stress state and a threshold value, respectively. If $r <\overline{r}$, the local state of stress is considered as isotropic, in the other case is considered anisotropic. According to our mechanobiological picture, an anisotropic stress state enhances transition towards the proliferative state, whereas an isotropic stress state enhances transition towards the ECM secreting state. We use a similar approach to quantify the concentration-mediated effect by introducing the indicator $H(c-c_{\text {thr}})$, $c_{\text {thr}}$ being a threshold concentration for cell activity. For notational brevity, we let $H_r:=H(r-\bar{r})$ and $H_c:=H(c-c_{\text {thr}})$.

The following exchange/production rate terms are considered:

- mitotic proliferation: we suppose cells in pool n proliferate at a rate given by
  7a $$\begin{aligned}&\phi _{\text {n}}(1-(\phi _{\text {n}}+\phi _{\text {v}}+ \phi _{\text {q}}+\phi _{{\text {ECM}}})) \dfrac{c}{K_{\text {sat}}+c} k_{\text {g}} \\&\quad = \phi _{\text {n}}\phi _{{\text {fl}}} \dfrac{c}{K_{\text {sat}}+c} k_{\text {g}}. \end{aligned}$$
  Relation (7a) is a phenomenological law in which the term $\phi _{\text {n}}\phi _{{\text {fl}}}$ keeps into account contact inhibition effects, while the term $\displaystyle \frac{c}{K_{\text {sat}} +c}k_{\text {g}}$ is a nutrient–dependent modulation (Monod law [21]), $K_{\text {sat}}$ being the half-saturation constant and $k_{\text {g}}$ the maximum growth rate, respectively;
- ECM production rate: we consider here glycosaminoglycan (GAG) as the main marker for ECM accumulation [47] and we assume a simple proportionality law between the total amount of ECM and the secretion rate of GAG. Following [47] we assume the ECM production rate to be given by
  7b $$\begin{aligned} \dfrac{\phi _{\text {v}}}{V_{{\text {cell}}}} \, c \,E\, k_{\text {GAG}} \max \left[ 0,1-\dfrac{\phi _{\text {ECM}}}{\phi _{\text {ECM,max}}}\right] . \end{aligned}$$
  In the above relation, $V_{{\text {cell}}}$ is the volume of a single cell, the constant of proportionality $E>1$ accounts for the heterogeneous composition of cartilagineous ECM (water for 70–80% of its wet weight, collagen fibrils for 10–15% and GAG for 5%) [14], *c* is oxygen concentration and $k_{GAG}$ a growth factor. The last term model the fact that ECM synthesis attains its maximum value when no extracellular matrix is present because more space is available for matrix production. Then, as soon as sythesized matrix accumulates at each point of the biomass construct, the available space diminuishes until $\phi _{\text {ECM}}$ reaches a maximum value $\phi _{\text {ECM,max}}$ and matrix secretion ceases. Notice that in the description of GAG secretion, we are assuming that at the initial time level, biomass is constituted by a uniform layer of cells and matrix (see [57] for a similar approach). This corresponds to neglecting the very initial phase where the seeded cells proliferate and “pave” the scaffold wall, and is consistent with the mathematical fact that a continuum-based approach does not enable to reproduce the subcellular mechanisms that regulate the early mitotic process. These latter processes should be more properly described by treating seeded cells as individual units that behave according to cellular automata schemes [16, 18, 27, 28].
- decay pathways: all cellular compartments may evolve into quiescent (absence of cell activity due to an insufficient oxygen intake [20, 67]) or apoptotic phases (cellular death). Quiescence occurs if nutrient concentration *c* falls below the critical level $c_{thr}$, whereas apoptotic phase is related to age dependent cell death [60]. The time rates of change between state $\alpha$ ($\alpha ={\text {n}},{\text {v}},{\text {q}}$) and the inactive states (quiescence or apoptosis) are $k_{\text {qui}}$ and $k_{\text {apo}}$, respectively;
- exchange rate between pools ${{\text {n}}\leftrightarrow {\text {q}}}$: a first contribution in the direction ${\text {n}}\rightarrow {\text {q}}$ is regulated by the mitotic characteristic inverse time constant $1/\tau _\mathrm{m}$ and take the form
  7c $$\begin{aligned} \mp \displaystyle \frac{\phi _n}{\tau _\mathrm{m}}. \end{aligned}$$
  A second contribution is regulated by the probability rate $\beta _{{\text {q}}\rightarrow {\text {n}}}$ that a cell in pool ${\text {q}}$ enters into pool ${\text {n}}$, enhanced by the mechanical factor $H_r$, giving the rate term
  7d $$\begin{aligned} \pm \phi _{{\text {q}}} \beta _{{\text {q}}\rightarrow {\text {n}}} H_r \end{aligned}$$
- exchange rate between pools ${{\text {v}}\leftrightarrow {\text {q}}}$: the probability rate $\beta _{{\text {v}}\rightarrow {\text {q}}}$ that a cell in pool ${\text {v}}$ enters into pool ${\text {q}}$ and the opposite for $\beta _{{\text {q}}\rightarrow {\text {n}}}$ are mediated by the mechanical terms $H_r$ and $1-H_r$, respectively, to signify that anisotropy favors proliferation while isotropy ECM secretion.

According to the exchange laws illustrated above, the production terms associated with cell populations are defined as:

8a $$\begin{aligned} Q_{\text {n}}&=-\dfrac{\phi _{\text {n}}}{\tau _{\text {m}}}+ \phi _{\text {q}}\beta _{{\text {q}}\rightarrow {\text {n}}} H_r \\&\quad+\phi _{\text {n}}\phi _{{\text {fl}}} \dfrac{c}{K_{\text {sat}}+c} k_{\text {g}} -k_{\text {qui}}\phi _{\text {n}}(1-H_c) \end{aligned}$$

8b $$\begin{aligned} Q_{\text {v}}&=-\phi _{\text {v}}\beta _{{\text {v}}\rightarrow {\text {q}}}H_r+ \phi _{\text {q}}\beta _{{\text {q}}\rightarrow {\text {v}}} \left( 1-H_r \right) \\&\quad -k_{\text {qui}}\phi _{\text {v}}(1-H_c) - k_{\text {apo}}\phi _{\text {v}} \end{aligned}$$

8c $$\begin{aligned} Q_{\text {q}}&=\dfrac{\phi _{\text {n}}}{\tau _{\text {m}}}- \phi _{\text {q}}\beta _{{\text {q}}\rightarrow {\text {n}}}H_r+\phi _{\text {v}}\beta _{{\text {v}}\rightarrow {\text {q}}}H_r \\&\quad -\phi _{\text {q}}\beta _{{\text {q}}\rightarrow {\text {v}}}\left( 1-H_r\right) -k_{\text {qui}}\phi _{\text {q}}\left( 1-H_c\right) - k_{\text {apo}}\phi _{\text {q}}. \end{aligned}$$

8d $$\begin{aligned} Q_{{\text {ECM}}} &=\dfrac{\phi _{\text {v}}}{V_{{\text {cell}}}} \, c \,E\, k_{\text {GAG}} \max \left[ 0,1-\dfrac{\phi _{\text {ECM}}}{\phi _{\text {ECM,max}}}\right] \\&\quad - k_{\text {deg}}\phi _{\text {ECM}}. \end{aligned}$$

To conclude the mathematical description of mass exchange terms, we define the extracellular fluid production $Q_{{\text {fl}}}$ in such a way to satisfy Assumption 3 and, consistently, relation (3d)

8e $$\begin{aligned} Q_{{\text {fl}}}=-\sum _{\eta ={\text {cells}}, {\text {ECM}}} Q_{\eta }. \end{aligned}$$

From a biophysical point of view this is equivalent to assuming that mass exchanges occur only among cells/ECM and fluid, meaning that dead cells and degrading ECM are deteriorated into extracellular fluid, and conversely that the latter is “consumed” whenever cells duplicate or secrete ECM [70]. From a computational point of view, relation (8e) allows us to eliminate the dependent variable $\phi _{{\text {fl}}}$ and the corresponding mass balance equation from system (3a) as done in [70], Sect. 2.2, in such a way that the fluid volume fraction can be computed by simple post-processing as

8f $$\begin{aligned} \phi _{{\text {fl}}} = 1 - \sum _{\eta ={\text {cells}}, {\text {ECM}}} \phi _{\eta }. \end{aligned}$$

## Bio-mechanical models for the deformable growing biomass

In this section we provide a mathematical description of the mechanobiological phenomena involving growth processes (cell duplication and ECM secretion). To this purpose, we introduce suitable bio-mechanical models for the growth tensors in the decomposition (2b) by extending the theory developed in [32] and [33].

### Growth laws

We propose the following definitions of the growth tensors:

9a $$\begin{aligned} {\varvec{\varepsilon }}^g_{\vartheta } (\mathbf{x },t; {\varvec{\phi }}) &=g_\theta ({\mathbf {x}},t; {\varvec{\phi }}){\mathbf {I}} \qquad \vartheta = {\text {v}}, {\text {q}}, {\text {ECM}} \end{aligned}$$

9b $$\begin{aligned} {\varvec{\varepsilon }}^g_{\text {n}}(\mathbf{x },t; {\varvec{\phi }}) &=g_{\text {n}}({\mathbf {x}},t; {\varvec{\phi }}){\mathbf {d}}^{{\text {pol}}} (\mathbf{x },t)\otimes {\mathbf {d}}^{{\text {pol}}} (\mathbf{x },t) \end{aligned}$$

where the symbol $\otimes$ represents the tensor dyadic product and $g_\theta$, $g_{\text {n}}$ are growth coefficients for which a model equation is provided below. Eqns. (9) state that the mass increment of each mixture solid constituent is isotropically deposited for the ${\text {v}}$, ${\text {q}}$ and ${\text {ECM}}$ components, while is accumulated along a specific polarization direction, identified by the unit vector ${\mathbf {d}}^{\text {pol}}$, for proliferating cells.

**Fig. 5:**
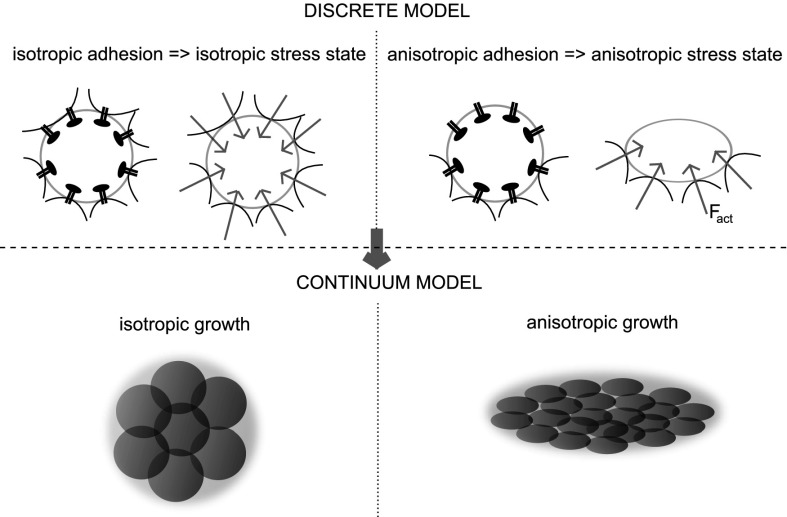
Cellular level (*top*): pictorial representation of the isotropic/anisotropic adherence condition. Continuum level (*bottom*): isotropic/anisotropic biomass growth

Biophysical motivations support our choice of the growth laws (9). Firstly, according to the concept of “force isotropy” on the cell introduced in [45, 46], cells that occupy the bio-synthesizing compartment (${\text {v}}$ compartment) experience an isotropic adherence condition and consequently tend to assume a spherical shape (see Fig. 5, left) whereas cells that live in the proliferating compartment (${\text {n}}$ compartment) are subjected to an anisotropic adhesion state and tend to elongate (see Fig. 5, right). Secondly, according to the infinitesimal deformation growth theory developed in [32], the deformation of an infinitesimal sphere of biomass growing into an ellipsoid can be reasonably described by an anisotropic growth tensor, while the deformation of an infinitesimal sphere growing into a larger sphere can be characterized by a isotropic growth tensor. For the sake of simplicity, the infinitesimal growth tensor for the species ${\text {q}}$ and ${\text {ECM}}$ are supposed to be isotropic. The definition of ${\mathbf {d}}^{\text {pol}}$ in Eq. (9b) and the law for its time evolution is a delicate issue. In [8], ${\mathbf {d}}^{\text {pol}}$ is characterized according to the dynamics of the evolution of the cell cytoskeleton, which reorganizes itself according to its mechano–sensing mechanisms. A simplified version of the model proposed in [8], and also adopted in the present work, is represented by the choice

9c $$\begin{aligned} {\mathbf {d}}^{\text {pol}}({\mathbf {x}},t)= {\mathbf {d}}_{\varepsilon } ({\mathbf {x}},t) \qquad \forall {\mathbf {x}} \in \varOmega , \quad \forall t>0 \end{aligned}$$

where ${\mathbf {d}}_{\varepsilon }$ is the normalized eigenvector of the infinitesimal strain tensor ${\varvec{\varepsilon }}_{{\text {s}}}$, associated with the eigenvalue of largest module, which physically corresponds to the maximum principal dilatation of the biomass around such a point [68].

The coefficients $g_{\eta }({\mathbf {x}},t; {\varvec{\phi }})$, $\eta = {\text {n}}, {\text {v}}, {\text {q}}, {\text {ECM}}$, give a measure of the amount of mass of the cellular population of type $\eta$ deposited at time *t* at point ${\mathbf {x}}$. To determine these quantities, we proceed as in [32] and [33] and require the following growth continuity initial value problem to be satisfied for all ${\mathbf {x}} \in \varOmega$ and for $\eta ={\text {cells}},{\text {ECM}}$:

9d $$\begin{aligned}\dfrac{\partial }{\partial t} {\text {Tr}}{}{\varvec{\varepsilon }}^{{\text {g}}}_{\eta } ({\mathbf {x}},t; {\varvec{\phi }}) = c_{R,\eta }({\mathbf {x}},t; {\varvec{\phi }}) \quad t \in (0, T_{end}] \end{aligned}$$

9e $$\begin{aligned}&{\text {Tr}}{}{\varvec{\varepsilon }}^{{\text {g}}}_{\eta } ({\mathbf {x}},0; {\varvec{\phi }}) = 0.&\end{aligned}$$

The quantity $c_{R,\eta }({\mathbf {x}},t; {\varvec{\phi }})$ represents the amount of mass of the cellular population $\eta$ deposited at time *t* at point ${\mathbf {x}}$ per unit time and per unit reference mass. According to the general indications illustrated in Sect. 2.2.4 of [33], the growth laws are phenomenological equations that indirectly describe chemical processes responsible for growth and can be typically expressed as “synthesis” rate minus a “degradation” rate, that may include a mass conversion rate from one constituent of the mixture to another. Also, the constants that appear in a specific growth law may depend parameterically on biological factors such as, for example, the level of a specific growth factor. Thus, based on the description carried out in Sect. 5, we set $c_{R,\eta }({\mathbf {x}},t; {\varvec{\phi }}):= Q_\eta ({\mathbf {x}},t; {\varvec{\phi }}), \, \eta ={\text {cells}},{\text {ECM}}$, in such a way that the initial value problems that furnish the characterization of the growth coefficients become, for $\theta ={\text {v}},{\text {q}}, {\text {ECM}}$:

9f $$\begin{aligned} \dfrac{\partial }{\partial t}g_\theta ({\mathbf {x}},t; {\varvec{\phi }}) = \displaystyle \frac{1}{3} Q_\theta ({\mathbf {x}},t; {\varvec{\phi }})\quad t \in (0, T_{end}] \end{aligned}$$

9g $$\begin{aligned}&g_\theta ({\mathbf {x}},t; {\varvec{\phi }}) = 0&\end{aligned}$$

and

9h $$\begin{aligned} \dfrac{\partial }{\partial t}g_{\text {n}}({\mathbf {x}},t; {\varvec{\phi }}) = Q_{\text {n}}({\mathbf {x}},t; {\varvec{\phi }})\quad t \in (0, T_{end}] \end{aligned}$$

9i $$\begin{aligned}&g_{\text {n}}({\mathbf {x}},t; {\varvec{\phi }}) = 0&\end{aligned}$$

having used the identities ${\text {Tr}}({\mathbf {I}}) = 3$ and ${\text {Tr}}({\mathbf {d}}^{\text {pol}}\otimes {\mathbf {d}}^{\text {pol}})=1$.

### Constitutive equations for the mechanical and fluidsubsystems

We assume that cells and ECM behave like linear elastic solids, so that the effective stress tensors associated with the solid components of the biomass are defined as

10a $$\begin{aligned} {\varvec{\sigma }}_{\eta }({\mathbf {u}}, \, \phi _{\eta }) &=2 \mu _\eta {\varvec{\varepsilon }}_{\eta }^{{\text {e}}}({\mathbf {u}}, \, \phi _{\eta }) + \lambda _\eta {\text {Tr}}{} {\varvec{\varepsilon }}_{\eta }^{\text {e}}({\mathbf {u}}, \, \phi _{\eta }) {\mathbf {I}} \\ &=2 \mu _\eta \left( {\varvec{\varepsilon }}({\mathbf {u}}) -{\varvec{\varepsilon }}_{\eta }^g(\phi _{\eta }) \right) + \lambda _\eta {\text {Tr}}{} \left( {\varvec{\varepsilon }}({\mathbf {u}}) -{\varvec{\varepsilon }}_{\eta }^g(\phi _{\eta })\right) {\mathbf {I}}, \end{aligned}$$

where $\lambda _{\eta }$ and $\mu _\eta$ are the Lamé parameters of each component of the solid phase, $\eta ={\text {n}}, {\text {q}}, {\text {v}}, {\text {ECM}}$, and ${\varvec{\varepsilon }}_{\eta }^g(\phi _{\eta })$ are the growth strain tensors introduced in (9). More sophisticated constitutive models might be adopted [3, 24, 42, 51], but their use is beyond the scope of the present work which is mainly devoted to proposing a computationally feasible mechanobiological model of *in vitro* cartilage tissue growth. We assume the relative velocity in Eq. (5c) to be expressed by the Darcy law (see, e.g., [11] and references cited therein)

10b $$\begin{aligned} \phi _{{\text {fl}}}{\mathbf {w}}=-{\mathbf {K}}(\phi _{\text {fl}})\nabla p \end{aligned}$$

where the isotropic permeability tensor ${\mathbf {K}}(\phi _{\text {fl}}) = \frac{\phi _{{\text {fl}}}^2}{C_{\text {F}}} {\mathbf {I}}$ is defined as in [65], $C_{\text {F}}$ being a friction coefficient. To provide a physically consistent characterization of $C_{\text {F}}$ we apply the classic Stokes theory for viscous drag to the biomass mixture and obtain

10c $$\begin{aligned} C_{\text {F}} = C_{\text {F,cell}} \phi _{{\text {s}}} = \frac{6 \pi \mu _{{\text {fl}}}}{A_{\text {cell}}} (1- \phi _{{\text {fl}}}) = \frac{3 \mu _{{\text {fl}}}}{2 R_{\text {cell}}^2} (1- \phi _{{\text {fl}}}) \end{aligned}$$

$R_{\text {cell}}$ and $\mu _{{\text {fl}}}$ being cell radius and interstitial fluid dynamic viscosity, respectively, from which we get

10d $$\begin{aligned} {\mathbf {K}}(\phi _{{\text {fl}}})= K_{\text {ref}} \frac{\phi _{{\text {fl}}}^2}{1- \phi _{{\text {fl}}}}{\mathbf {I}}, \qquad K_{\text {ref}} = \frac{2}{3} \frac{R_{cell}^2}{\mu _{{\text {fl}}}}. \end{aligned}$$

## The mathematical model in 3D

**Fig. 6:**
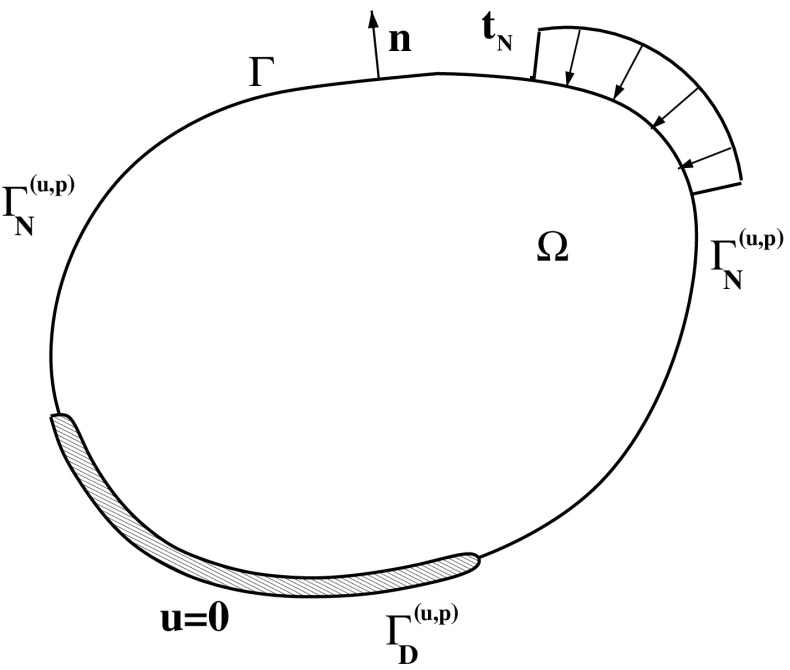
Schematic representation of the 3D computational domain. The boundary conditions for the mechanical block of the model are considered. In this example, ${\mathbf {u}}_D = {\mathbf {0}}$ whereas ${\mathbf {t}}_N$ is nonvanishining only on a subset of $\varGamma _N^{({\mathbf {u}}, p)}$

In this section we summarize the mathematical model for the multiphase mixture constituting the growing tissue. We refer to Table 1 for the definition of all model parameters and their quantitative value used in numerical simulations. In what follows, we denote by $\varOmega$ an open bounded set of ${\mathbb {R}}^3$ representing the computational domain in which the biomass growth process physically takes place, and by $\varGamma :=\partial \varOmega$ the boundary of $\varOmega$ on which an outward unit normal vector ${\mathbf {n}}$ is defined (see Fig. 6). In view of the definition of the boundary conditions to be supplied to the mechanobiological model, it is convenient to indicate by ${\mathcal {U}}: = \left\{ ({\mathbf {u}},p), c, {\varvec{\phi }} \right\}$ the set of the dependent variables of the problem. Then, for each $\mathcal {u} \in {\mathcal {U}}$ we assume that $\varGamma$ is divided into two disjoint portions, denoted by $\varGamma _D^{\mathcal {u}}$ and $\varGamma _N^{\mathcal {u}}$, such that $\varGamma = \varGamma _D^{\mathcal {u}} \cup \varGamma _N^{\mathcal {u}}$, and where Dirichlet and Neumann boundary conditions are applied, respectively. By doing so, if $\mathcal {u}=({\mathbf {u}},p)$ the boundary conditions are for the model block of Sect. 4.3, if $\mathcal {u}=c$ the boundary conditions are for the model block of Sect. 4.4 whereas if $\mathcal {u}={\varvec{\phi }}$ the boundary conditions are for the model block of Sect. 4.1. We notice that, in general, there is no geometrical relation between the various decompositions of the domain boundary belonging to the same typology of boundary condition. The sole requirement is that, for each considered dependent variable, the union of the Dirichlet and Neumann partitions is the whole domain boundary $\varGamma$. For a similar treatment of the splitting of the domain boundary we refer to [26] and [7].

Having specified the geometrical setting of the biomass growth process, the mathematical model proposed in the present article consists of the following PDE subsystems:

*Poroelastic IV-BVP for biomass:* for given ${\varvec{\phi }}={\varvec{\phi }}({\mathbf {x}},t)$, find the solid displacement ${\mathbf {u}}$, the fluid friction velocity ${\mathbf {w}}$ and pressure *p* such that the following equations are satisfied in ${\mathcal {Q}}_{T_{end}}$:

11a $$\begin{aligned}&\displaystyle \frac{\partial }{\partial t}{\text {div}}{} {\mathbf {u}} + {\mathrm {div}}\left( \phi _{{\text {fl}}} {\mathbf {w}}\right) = 0&\end{aligned}$$

11b $$\begin{aligned}&{\mathbf {div}}{}{\mathbf {T}}({\mathbf {u}}, p,{\varvec{\phi }}) = {\mathbf {0}}&\end{aligned}$$

11c $$\begin{aligned}&\phi _{{\text {fl}}}{\mathbf {w}}=-{\mathbf {K}}(\phi _{\text {fl}})\nabla p, \qquad {\mathbf {K}}(\phi _{\text {fl}})=K_{\text {ref}}\displaystyle \frac{\phi _{{\text {fl}}}^2}{1-\phi _{{\text {fl}}}}{\mathbf {I}}&\end{aligned}$$

11d $$\begin{aligned}&{\mathbf {T}}({\mathbf {u}}, p,{\varvec{\phi }}) = \sum _{\eta ={\text {cells}},{\text {ECM}}} \phi _\eta {\varvec{\sigma }}_\eta ({\mathbf {u}},{\varvec{\phi }}) - p {\mathbf {I}}&\end{aligned}$$

11e $$\begin{aligned}&\sigma _\eta ({\mathbf {u}},{\varvec{\phi }})= 2 \mu _\eta ({\varvec{\varepsilon }}({\mathbf {u}}) - {\varvec{\varepsilon }}^{g}_\eta (\phi _\eta ))+ \lambda _\eta {\text {Tr}}{} ({\varvec{\varepsilon }}({\mathbf {u}})- {\varvec{\varepsilon }}^{g}_\eta (\phi _\eta )) {\mathbf {I}}&\end{aligned}$$

11f $$\begin{aligned}&{\varvec{\varepsilon }}({\mathbf {u}}) = \frac{1}{2} (\nabla {\mathbf {u}} + (\nabla {\mathbf {u}})^T)&\end{aligned}$$

11g $$\begin{aligned}&\displaystyle \frac{\partial }{\partial t}g_{\text {n}}({\mathbf {x}},t;{\varvec{\phi }}) = Q_{\text {n}}({\mathbf {x}},t; {\varvec{\phi }})&\end{aligned}$$

11h $$\begin{aligned}&\displaystyle \frac{\partial }{\partial t}g_\theta ({\mathbf {x}},t;{\varvec{\phi }}) = \displaystyle \frac{1}{3}Q_{\text {n}}({\mathbf {x}},t; {\varvec{\phi }})&\end{aligned}$$

supplied with the following initial and boundary conditions:

11i $$\begin{aligned}&{\mathbf {u}}({\mathbf {x}},0)={\mathbf {u}}^{0}({\mathbf {x}}) \qquad {\text {in }}\,\,\varOmega&\end{aligned}$$

11j $$\begin{aligned}&{\mathbf {u}}({\mathbf {x}},t)={\mathbf {u}}_D({\mathbf {x}},t) \qquad ({\mathbf {x}},t) \in \left( \varGamma _D^{({\mathbf {u}},p)} \times (0, T_{end}) \right)&\end{aligned}$$

11k $$\begin{aligned}&{\mathbf {T}}({\mathbf {u}}, p,{\varvec{\phi }}) {\mathbf {n}}({\mathbf {x}},t)={\mathbf {t}}_N({\mathbf {x}},t) \qquad ({\mathbf {x}},t) \in \left( \varGamma _N^{({\mathbf {u}},p)} \times (0, T_{end}) \right)&\end{aligned}$$

where ${\mathbf {u}}^{0}: \varOmega \rightarrow {\mathbb {R}}^3$ is the initial mixture displacement in the domain whereas ${\mathbf {u}}_D : \left( \varGamma _D^{({\mathbf {u}},p)} \times (0, T_{end}) \right) \rightarrow {\mathbb {R}}^3$ and ${\mathbf {t}}_N : \left( \varGamma _N^{({\mathbf {u}},p)} \times (0, T_{end}) \right) \rightarrow {\mathbb {R}}^3$ are the given displacement and traction fields defined on the decomposition $\varGamma _D^{({\mathbf {u}},p)} \cup \varGamma _N^{({\mathbf {u}},p)}$ of the computational domain boundary in the mechanical block of the model.

*Mass balance IV-BVP for nutrient concentration:* for given ${\mathbf {w}}={\mathbf {w}}({\mathbf {x}},t)$, ${\mathbf {u}} = {\mathbf {u}}({\mathbf {x}},t)$ and ${\varvec{\phi }}={\varvec{\phi }}({\mathbf {x}},t)$, find the nutrient concentration *c* and the nutrient flux density ${\mathbf {J}}_c$ such that the following equations are satisfied in ${\mathcal {Q}}_{T_{end}}$:

12a $$\begin{aligned} \frac{\partial c}{\partial t}+ {\text {div}}\,{\mathbf {J}}_c &=Q_c({\varvec{\phi }}, c) \end{aligned}$$

12b $$\begin{aligned} {\mathbf {J}}_c &={\mathbf {v}}_{{\text {fl}}} c -D_c \nabla c \end{aligned}$$

12c $$\begin{aligned} {\mathbf {v}}_{{\text {fl}}} &={\mathbf {w}} + \dfrac{\partial {\mathbf {u}}}{\partial t} \end{aligned}$$

12d $$\begin{aligned} {Q}_c({\varvec{\phi }},c) &=-(R_{\text {n}}\phi _{\text {n}}+R_{\text {v}}\phi _{\text {v}}+R_{\text {q}}\phi _{\text {q}})\frac{c}{c+K_{1/2}} \end{aligned}$$

supplied with the following initial and boundary conditions:

12e $$\begin{aligned}&c({\mathbf {x}},0) = c^0({\mathbf {x}}) \qquad {\text {in }}\,\,\varOmega&\end{aligned}$$

12f $$\begin{aligned}&c({\mathbf {x}},t) = c_D({\mathbf {x}},t) \qquad ({\mathbf {x}},t) \in \left( \varGamma _D^{c} \times (0, T_{end}) \right)&\end{aligned}$$

12g $$\begin{aligned}&- D_c \nabla c \cdot {\mathbf {n}}({\mathbf {x}},t)=g_N({\mathbf {x}},t) \qquad ({\mathbf {x}},t) \in \left( \varGamma _N^{c} \times (0, T_{end}) \right)&\end{aligned}$$

where $c^{0}: \varOmega \rightarrow R^+$ is the initial nutrient concentration in the domain whereas $c_D : \left( \varGamma _D^{c} \times (0, T_{end}) \right) \rightarrow {\mathbb {R}}$ and $g_N : \left( \varGamma _N^{c} \times (0, T_{end}) \right) \rightarrow {\mathbb {R}}$ are the given concentration and diffusive nutrient flux defined on the decomposition $\varGamma _D^{c} \cup \varGamma _N^{c}$ of the computational domain boundary in the nutrient block of the model.

*Mass conservation IV-BVP for cellular populations:* for given ${\varvec{\phi }}={\varvec{\phi }}({\mathbf {x}},t)$, $c=c({\mathbf {x}},t)$, ${\mathbf {T}}={\mathbf {T}}({\mathbf {x}},t)$ and ${\mathbf {u}} = {\mathbf {u}}({\mathbf {x}},t)$, find the volume fractions ${\varvec{\phi }}$ such that the following equations are satisfied in ${\mathcal {Q}}_{T_{end}}$:

13a $$\begin{aligned} \frac{\partial {\varvec{\phi }}}{\partial t}+ {\mathbf {div}}\,{\mathbf {J}}_{{\varvec{\phi }}} &={\mathbf {Q}}({\varvec{\phi }},c,{\mathbf {T}}) \end{aligned}$$

13b $$\begin{aligned} {\mathbf {J}}_{{\varvec{\phi }}} &=\left[ \phi _{\text {fl}}{\mathbf {v}}_{{\text {s}}},\phi _{\text {n}}{\mathbf {v}}_{{\text {s}}}, \phi _{\text {v}}{\mathbf {v}}_{{\text {s}}}, \phi _{\text {q}}{\mathbf {v}}_{{\text {s}}},\phi _{{\text {ECM}}} {\mathbf {v}}_{{\text {s}}}\right] ^T \end{aligned}$$

13c $$\begin{aligned} {\mathbf {v}}_{{\text {s}}} &=\displaystyle \frac{\partial {\mathbf {u}}}{\partial t} \end{aligned}$$

13d $$\begin{aligned} \phi _{{\text {fl}}} &=1 - \phi _{\text {s}} \end{aligned}$$

where $${\mathbf {Q}}=[Q_{\text {fl}},Q_{\text {n}},Q_{\text {v}},Q_{\text {q}},Q_{\text {ECM}}]^T$$, with

13e $$\begin{aligned} Q_{\text {n}}&=-\dfrac{\phi _{\text {n}}}{\tau _{\text {m}}}+ \phi _{\text {q}}\beta _{{\text {q}}\rightarrow {\text {n}}} H_r \\&+\phi _{\text {n}}\phi _{{\text {fl}}} \dfrac{c}{K_{\text {sat}}+c} k_{\text {g}} -k_{\text {qui}}\phi _{\text {n}}(1-H_c) \end{aligned}$$

13f $$\begin{aligned} Q_{\text {v}}&=-\phi _{\text {v}}\beta _{{\text {v}}\rightarrow {\text {q}}}H_r+ \phi _{\text {q}}\beta _{{\text {q}}\rightarrow {\text {v}}} \left( 1-H_r \right) \\&-k_{\text {qui}}\phi _{\text {v}}(1-H_c) - k_{\text {apo}}\phi _{\text {v}} \end{aligned}$$

13g $$\begin{aligned} Q_{\text {q}}&=\dfrac{\phi _{\text {n}}}{\tau _{\text {m}}}- \phi _{\text {q}}\beta _{{\text {q}}\rightarrow {\text {n}}}H_r+\phi _{\text {v}}\beta _{{\text {v}}\rightarrow {\text {q}}}H_r \\&-\phi _{\text {q}}\beta _{{\text {q}}\rightarrow {\text {v}}}\left( 1-H_r\right) -k_{\text {qui}}\phi _{\text {q}}\left( 1-H_c\right) - k_{\text {apo}}\phi _{\text {q}}. \end{aligned}$$

13h $$\begin{aligned} Q_{{\text {ECM}}} &=\dfrac{\phi _{\text {v}}}{V_{{\text {cell}}}} \, c \,E\, k_{\text {GAG}} \max \left[ 0,1-\dfrac{\phi _{\text {ECM}}}{\phi _{\text {ECM,max}}}\right] \\&- k_{\text {deg}}\phi _{\text {ECM}} \end{aligned}$$

supplied with the following initial and boundary conditions:

13i $$\begin{aligned} {\varvec{\phi }}({\mathbf {x}},0)&={\varvec{\phi }}^0({\mathbf {x}}) \qquad {\text {in }}\,\,\varOmega&\end{aligned}$$

13j $$\begin{aligned} {\mathbf {J}}_{{\varvec{\phi }}} {\mathbf {n}} ({\mathbf {x}},t)&={\mathbf {0}} \qquad ({\mathbf {x}},t) \in \left( \varGamma \times (0, T_{end}) \right)&\end{aligned}$$

where ${\varvec{\phi }}^0 : \varOmega \rightarrow ({\mathbb {R}}^+)^5$ is the initial distribution of cellular volume fraction in the domain. Condition (13j) amounts to assuming that no cellular flux is exchanged with the external environment during the biomass growth process.

## The mechanobiological model in 1D

**Fig. 7:**
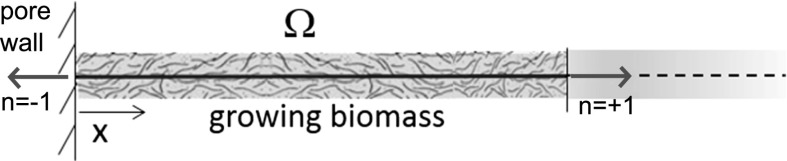
Schematic representation of the 1D scaffold-based bioreactor

In this section we formulate the proposed mechanobiological model in a one-dimensional (1D) geometrical configuration. This is a first step toward the simulation of a realistic structure such as the 3D scaffolded bioreactor used in the experimental analysis discussed in [34]. The 1D-formulation is constructed by describing the biomass as a nonhomogeneous bar (fixed at one endpoint) subject to a uniaxial state of mechanical stress in such a way that each point of the bar undergoes the *same* deformation. Then we consider the following assumptions:

- all model variables depend on the sole spatial coordinate *x* and on the time variable *t*;
- the solid displacement field ${\mathbf {u}}$ has only one nonvanishing component, that is ${\mathbf {u}} = [u, 0, 0]^T$ with $u=u(x,t)$;
- the strain tensor has only one nonvanishing component, that is $\varepsilon _{xx}({\mathbf {u}},t) = \partial u(x,t) /\partial x$;

Figure 7 shows the computational domain. The region $x < 0$ represents the scaffold wall, the open interval $\varOmega = (0, L)$ is the growing tissue whereas the region $x > L$ corresponds to the interstitial fluid that brings nutrient to the growing construct. We denote by $\varGamma := \partial \varOmega = \left\{ 0, \, L \right\}$ the boundary of the computational domain and by *n* the outward unit normal vector on $\partial \varOmega$. We have ${\mathbf {n}}=-1$ at $x=0$ and ${\mathbf {n}}=+1$ at $x=L$.

The 1D mechanobiological model consists of the following PDE subsystems:

*Poroelastic IV-BVP for biomass* for given $\phi _\eta$ and $g_\eta$, $\eta ={\text {cells}}, {\text {ECM}}, {\text {fl}}$, find the solid displacement $u:{\mathcal {Q}}_{T_{end}} \rightarrow {\mathbb {R}}$, the fluid friction velocity $w:{\mathcal {Q}}_{T_{end}}\rightarrow {\mathbb {R}}$ and the pressure $p:{\mathcal {Q}}_{T_{end}}\rightarrow {\mathbb {R}}$ that satisfy the following system of partial differential equations in balance form:

14a $$\begin{aligned} \displaystyle \frac{\partial T_{xx}}{\partial x} &=0 \end{aligned}$$

14b $$\begin{aligned} \displaystyle \frac{\partial }{\partial t}\displaystyle \frac{\partial u}{\partial x} + \displaystyle \frac{\partial V}{\partial x} &=0 \end{aligned}$$

14c $$\begin{aligned} V&=-K(\phi _{\text {fl}})\frac{\partial p}{\partial x} \end{aligned}$$

14d $$\begin{aligned} T_{xx} &=H_{\mathrm {A}}\phi _{\text {s}}\displaystyle \frac{\partial u}{\partial x} - p - H_{\mathrm {A}}\phi _{\text {n}}g_n - H_{\mathrm {B}}\sum _{\eta ={\text {v}},{\text {q}},{\text {ECM}}} \phi _{\eta }g_\eta \end{aligned}$$

where

14e $$\begin{aligned}&K(\phi _{\text {fl}})=K_{\text {ref}}\displaystyle \frac{\phi _{\text {fl}}^2}{1-\phi _{\text {fl}}}&\end{aligned}$$

is the tissue permeability with $K_{\text {ref}}$ defined in Eq. (10d) (right) whereas $H_{\mathrm {A}}=\lambda +2\mu$ is the so-called aggregate modulus [69] with $H_{\mathrm {B}}=3\lambda +2\mu$. To close the problem, we specify the following initial and boundary conditions:

14f $$\begin{aligned}&u(x,0)=u^{0}(x)&\qquad {\text {in}}\quad \, \varOmega&\end{aligned}$$

14g $$\begin{aligned}&u(0,t)=0&\qquad \forall t \in (0,T)&\end{aligned}$$

14h $$\begin{aligned}&T_{xx}(L,t) \cdot {\mathbf {n}} = T_{\text {b}}(t)&\qquad \forall t \in (0,T)&\end{aligned}$$

14i $$\begin{aligned}&p(0,t)=0&\qquad \forall t \in (0,T)&\end{aligned}$$

14j $$\begin{aligned}&V(L,t) \cdot {\mathbf {n}} = V_{\text {b}}(t)&\qquad \forall t \in (0,T).&\end{aligned}$$

*Mass balance IV-BVP for nutrient concentration* for given *u*, *V* and $\phi _\eta$, $\eta = {\text {n}}, {\text {v}}, {\text {q}}, {\text {fl}}$, find the oxygen nutrient concentration $c: {\mathcal {Q}}_{T_{end}} \rightarrow {\mathbb {R}}^+$ that satisfies the following system of partial differential equations in balance form:

15a $$\begin{aligned}&\frac{\partial c}{\partial t}+ \frac{\partial J_{\mathrm {c}}}{\partial x}= Q_{\mathrm {c}}(\widetilde{{\varvec{\phi }}}, c)&\end{aligned}$$

15b $$\begin{aligned}&J_c = v_{{\text {fl}}} c-D_{\mathrm {c}} \frac{\partial c}{\partial x}&\end{aligned}$$

where:

15c $$\begin{aligned}&w=\frac{V}{\phi _{\text {fl}}}&\end{aligned}$$

15d $$\begin{aligned}&v_{{\text {fl}}} = w + \dfrac{\partial u}{\partial t}&\end{aligned}$$

and

15e $$\begin{aligned}&D_{\mathrm {c}}=D_{{\mathrm {c}},{\text {fl}}}\dfrac{3k-2\phi _{\text {fl}}(k-1)}{3+\phi _{\text {fl}}(k-1)}, \qquad k:=K_{\mathrm {eq}}\dfrac{D_{{\mathrm {c}},{\text {s}}}}{D_{{\mathrm {c}},{\text {fl}}}}&\end{aligned}$$

15f $$\begin{aligned}&Q_{\mathrm {c}}(\widetilde{{\varvec{\phi }}},c) = - (R_{\text {n}}\phi _{\text {n}}+ R_{\text {v}}\phi _{\text {v}}+ R_{\text {q}}\phi _{\text {q}}) \displaystyle \frac{c}{c+K_{1/2}}.&\end{aligned}$$

To close the problem, we specify the following initial and boundary conditions:

15g $$\begin{aligned}&c(x,0) = c^0(x)&\qquad {\text {in }} \varOmega&\end{aligned}$$

15h $$\begin{aligned}&\left. \dfrac{\partial c}{\partial x}\right| _{x=0}=0&\qquad \forall \,t&\end{aligned}$$

15i $$\begin{aligned}&c(L,t)=c_{\text {ext}}(t).&\end{aligned}$$

*Mass conservation IV-BVP for cellular populations* for given *u*, *p* and *c*, find the volume fractions

16 $$\begin{aligned} \widetilde{{\varvec{\phi }}} = \left[ \phi _{\text {n}}, \, \phi _{\text {v}}, \, \phi _{\text {q}}, \, \phi _{{\text {ECM}}} \right] ^T : ({\mathcal {Q}}_{T_{end}})^4 \times ({\mathbb {R}}^+)^4 \end{aligned}$$

and $\phi _{{\text {fl}}}: {\mathcal {Q}}_{T_{end}} \rightarrow {\mathbb {R}}^+$ that satisfy the following system of partial differential equations in balance form:

17a $$\begin{aligned}&\frac{\partial \widetilde{{\varvec{\phi }}}}{\partial t}+ \displaystyle \frac{\partial J_{\widetilde{{\varvec{\phi }}}}}{\partial x}= {\mathbf {Q}}(\widetilde{{\varvec{\phi }}},c, {\mathbf {T}})= ({\mathbf {P}}(\widetilde{{\varvec{\phi }}},c, {\mathbf {T}})-{\mathbf {C}}(c,{\mathbf {T}}))\widetilde{{\varvec{\phi }}}&\end{aligned}$$

17b $$\begin{aligned}&\left( J_{\widetilde{{\varvec{\phi }}}}\right) _\eta = \phi _\eta v_{{\text {s}}}-D_\eta \displaystyle \frac{\partial \phi _\eta }{\partial x} \qquad \eta = {\text {cells}}, {\text {ECM}}&\end{aligned}$$

where $v_{{\text {s}}} = \partial u/\partial t$ is the solid phase velocity, the production terms are:

17c $$\begin{aligned}&{\mathbf {P}}({\varvec{\phi }},c,{\mathbf {T}}) = \end{aligned}$$

17d $$\begin{aligned}&\left[ \phi _{\text {fl}}\dfrac{c}{K_{\text {sat}}+c} k_{\text {g}},0, \beta _{{\text {q}}\rightarrow {\text {n}}}H_r, 0;\quad 0,0,\beta _{{\text {q}}\rightarrow {\text {v}}}\left( 1-H_r\right) ,0;\right. \\&\left. \dfrac{1}{\tau _{\text {m}}},\beta _{{\text {v}}\rightarrow {\text {q}}}H_r,0,0;\quad 0,\dfrac{1}{V_{{\text {cell}}}} \, c \,E\, k_{\text {GAG}} \max \left[ 0,1-\dfrac{\phi _{\text {ECM}}}{\phi _{\text {ECM,max}}}\right] , 0,0\right] \end{aligned}$$

17e $$\begin{aligned}&{\mathbf {C}}(c,{\mathbf {T}}) = \end{aligned}$$

17f $$\begin{aligned}&{\mathrm {diag}}(\dfrac{1}{\tau _{\text {m}}}+k_{\text {qui}}(1-H_c),\; \beta _{{\text {v}}\rightarrow {\text {q}}}H_r+k_{\text {qui}}(1-H_c)+ k_{\text {apo}}, \\&\beta _{{\text {q}}\rightarrow {\text {n}}}H_r+ \beta _{{\text {q}}\rightarrow {\text {v}}}\left( 1-H_r \right) +k_{\text {qui}}\left( 1-H_c\right) + k_{\text {apo}},\; k_{\text {deg}}). \end{aligned}$$

and the fluid fraction is computed as

17g $$\begin{aligned}&\phi _{{\text {fl}}} = 1 - \sum _{\eta ={\text {n}}, {\text {v}}, {\text {q}}, {\text {ECM}}} \phi _{\eta }.&\end{aligned}$$

To close the problem, we specify the following initial and boundary conditions:

17h $$\begin{aligned}&\widetilde{{\varvec{\phi }}}(x,0)=\widetilde{{\varvec{\phi }}}^0(x)&\qquad {\text {in }} \varOmega&\end{aligned}$$

17i $$\begin{aligned}&\left. \dfrac{\partial \phi _\eta }{\partial x}\right| _{x=0}=0&\quad \qquad \forall \,t\qquad \eta ={\text {n}},{\text {v}},{\text {q}},{\text {ECM}}&\end{aligned}$$

17j $$\begin{aligned}&\left. \dfrac{\partial \phi _\eta }{\partial x}\right| _{x=L}=0&\qquad \forall \,t\qquad \eta ={\text {n}},{\text {v}},{\text {q}},{\text {ECM}}.&\end{aligned}$$

The boundary conditions (17i)- (17j) express the fact that cellular phases can flow out of the biomass only because of the presence of an advective field.

### Indicator of the isotropy of the local stress state

In the 1D configuration, the total stress tensor ${\mathbf {T}}$ can be decomposed into the sum of isotropic and anisotropic components ${\mathbf {T}} = {\mathbf {T}}_{\text {iso}} + {\mathbf {T}}_{\text {aniso}}$ given by:

18a $$\begin{aligned} {\mathbf {T}}_{\text {iso}}&=\lambda \left( \phi _{\text {s}}\dfrac{\partial u}{\partial x}- g_{\text {n}}\phi _{\text {n}}\right) {\mathbf {I}} \\&\quad -\left( \dfrac{2}{3}\mu +\lambda \right) \sum _{\eta ={\text {v}},{\text {q}},{\text {ECM}}}g_{\eta }\phi _\eta {\mathbf {I}} -p {\mathbf {I}} \end{aligned}$$

18b $$\begin{aligned} {\mathbf {T}}_{\text {aniso}}&=2\mu \left( \phi _{\text {s}}\dfrac{\partial u}{\partial x}-g_{\text {n}}\phi _{\text {n}}\right) {\mathbf {d}}_{\text {pol}}\otimes {\mathbf {d}}_{\text {pol}}, \end{aligned}$$

where ${\mathbf {d}}_{\text {pol}}$ is the unit vector $[1,0,0]^T$. We use ${\mathbf {T}}_{\text {aniso}}$ to measure the degree of anisotropicity of the stress state at any point *x* of the mixture and at any time *t*. Namely, we define the parameter *r* as

18c $$\begin{aligned} r(x,t)&=\displaystyle \frac{\Vert {\mathbf {T}}_{\text {aniso}}(x,t)\Vert _F}{2 \mu } \\&= \left| \phi _{\text {s}}(x,t) \displaystyle \frac{\partial u(x,t)}{\partial x}-g_{\text {n}}(x,t) \phi _{\text {n}}(x,t)\right| \end{aligned}$$

where $\Vert \cdot \Vert _F$ is the Frobenius norm. We can give a mechanical interpretation of (18c) by studying the Mohr circle at point (*x*, *t*). The principal components of ${\mathbf {T}}$ are:

18d $$\begin{aligned} \sigma _{\text {I}}&=H_{\text {A}}\phi _{\text {s}}\dfrac{\partial u}{\partial x}-p-H_{\text {A}}g_{\text {n}}\phi _{\text {n}}-H_{\text {B}} \sum _{\eta ={\text {v}},{\text {q}},{\text {ECM}}}g_{\eta }\phi _\eta \end{aligned}$$

18e $$\begin{aligned} \sigma _{\text {II}}&=\sigma _{\text {III}}=\lambda \phi _{\text {s}}\dfrac{\partial u}{\partial x}-p-\lambda g_{\text {n}}\phi _{\text {n}}-H_{\text {B}}\sum _{\eta ={\text {v}},{\text {q}},{\text {ECM}}}g_{\eta }\phi _\eta , \end{aligned}$$

from which it follows that the Mohr circle at (*x*, *t*) has center $C=(\sigma _{\text {I}}+\sigma _{\text {II}})/2$ and radius equal to the maximum total shear stress at (*x*, *t*)

$$\begin{aligned} \tau _{\text {max}}(x,t) &=\dfrac{\sigma _{\text {I}}(x,t) - \sigma _{\text {II}}(x,t)}{2} \\&= \mu \left( \phi _{\text {s}}(x,t) \displaystyle \frac{\partial u(x,t)}{\partial x}-g_{\text {n}}(x,t) \phi _{\text {n}}(x,t) \right) . \end{aligned}$$

Comparing the latter relation with (18c) we conclude that the indicator of the local stress state anisotropy can be written as

18f $$\begin{aligned} r(x,t) = \displaystyle \frac{|\tau _{\text {max}}(x,t)|}{\mu }. \end{aligned}$$

We also need to characterize an appropriate value for the threshold parameter $\bar{r}$ representing the level of hydrodynamic shear stress that induces metabolic activity of the cell population ${\text {n}}$ and therefore separates the isotropic regime from the anisotropic regime. In [57, 58] it is shown that hydrodynamic shear below 10 mPa may promote GAG synthesis, so that, coherently with (18f), we assume

18g $$\begin{aligned} \bar{r}=\dfrac{{\mathrm {10mPa}}}{\mu }. \end{aligned}$$

## Numerical approximation of the 1D mechanobiological model

Solving in closed form the mechanobiological model proposed in this article is a very difficult task because of the strong nonlinear nature of the problem. Therefore, in this section we illustrate the approximation methods that are used to solve numerically the equation system in the 1D setting of Fig. 7.

### Computational algorithm

**Fig. 8:**
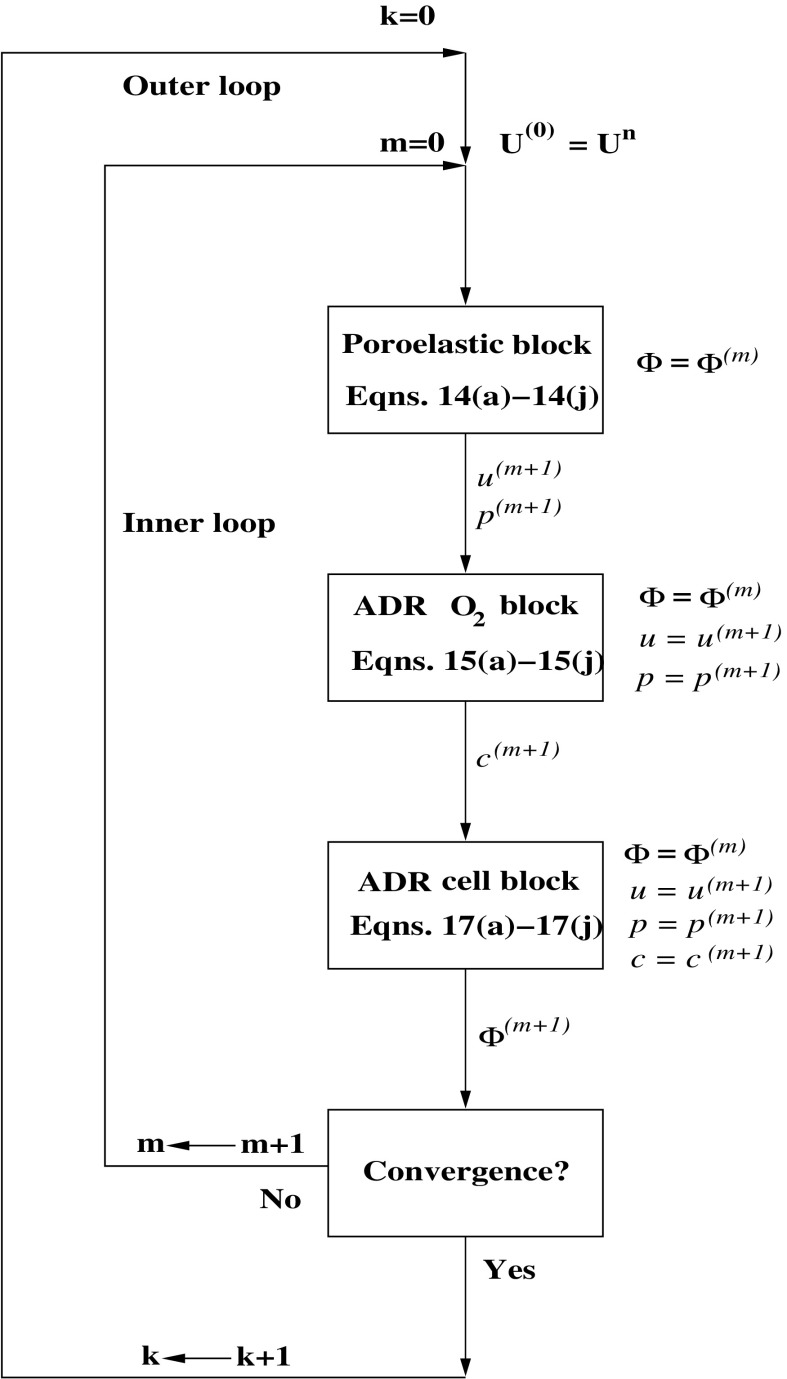
Solution map

Prior to discretization, we need to reduce the solution of the whole coupled system to the solution of a sequence of linearized equations of simpler form. For this purpose we set

19a $$\begin{aligned}&{\mathbf {U}} = \left[ u, p, \phi _{\text {n}}, \phi _{\text {v}}, \phi _{\text {q}}, \phi _{\text {ECM}}, c \right] ^T&\end{aligned}$$

and subdivide the time interval $[0, T_{end}]$ into $N_T \ge 1$ uniform subintervals of length $\varDelta t = T_{end}/N_T$, in such a way that the discrete time levels $t^k= k \varDelta t$, $k = 0, \ldots , N_T$, are obtained. Then, for each $k = 0, \ldots , N_T-1$, we set ${\mathbf {U}}^{(0)}:={\mathbf {U}}^n$ and for all $m \ge 0$ until convergence we perform the fixed point iteration schematically illustrated in Fig. 8. The algorithm consusts of two nested loops, a temporal outer loop and a spatial inner loop. For each step *m* of the inner loop we solve in sequence three linear PDE system blocks. For the convenience of the reader, for each substep of the inner loop ,we indicate in Fig. 8 the equations that are solved in the step referring to their numbering in Sect. 8. In order, the subproblems to be solved are: a poroelastic system for biomass displacement and fluid pressure, an advection–diffusion–reaction (ADR) system for oxygen concentration and an ADR system for cellular populations and ECM. For each system we indicate on the right of the corresponding block the values of the dependent variables that are *given inputs* whereas the updated values of the dependent variables that are returned as *outputs* of the block are indicated on the right of the downward arrow that exits out the block. We notice that as soon as a newly updated variable is available such variable is immediately plugged into the successive block as input variable. For this reason the algorithm of Fig. 8 can be regarded as a nonlinear block Gauss-Seidel method (see [52] Chapt. 7).

Two remarks are in order about the above described solution map. The first remark concerns the linear poroelastic system. The weak formulation of this problem leads to solving a saddle-point problem in block symmetric form to which the abstract analysis of [53], Chapt. 7 and [9] can be applied to prove existence and uniqueness of the solution pair $u^{(m+1)}, \, p^{(m+1)}$. The second remark concerns with the two linear ADR problems to which the application of the maximum principle (see [59]) allows to prove nonnegativity of the solutions $c^{(m+1)}$ and $\phi _\eta ^{(m+1)}$, $\eta = {\text {cells}}, {\text {ECM}}$.

### Finite element discretization

The computational procedure described in Sect. 9.1 leads to solving two kinds of BVPs: (1) a saddle-point problem; (2) two ADR equations. We numerically solve (1) and (2) using the Galerkin finite element approximation scheme on a family of partitions $\left\{ {\mathcal {T}}_h \right\} _{h>0}$ of the computational domain, *h* being the discretization parameter (see [53]). In the case of the saddle point problem (1) we employ piecewise linear finite elements on ${\mathcal {T}}_h$ for both solid displacement and fluid pressure. Equal-order interpolation for *u* and *p* does not give rise to numerical instabilities as it would be the case if the Stokes equations for an incompressible fluid were to be solved (cf. [53], Chapt. 9), because in the present model the variable *p* is *not* a Lagrange multiplier (as in the Stokes system), rather, it is the solution of an elliptic Darcy problem (for a similar treatment see [12]). In the case of the ADR equation we employ for the approximation of the concentration and of the cellular volume fractions the primal-mixed finite element discretization scheme with exponential fitting stabilization proposed and investigated in [62]. This choice is taken because it ensures that the computed numerical solutions satisfy a strict positivity property even in the case of a strongly advective regime. Moreover, it can be checked that, if advective terms do not play a major role compared to oxygen molecular diffusion in the biomass, then the effect of the stabilization introduced by the primal-mixed method of [62] becomes negligible so that the accuracy of the scheme is not spoiled. This, instead, would not be the case if the classic upwind stabilization were adopted (see [10] for a discussion of this important issue).

## Simulation tests

In this section we show the numerical results obtained by solving the 1D problem with the computational algorithm described in Sect. 9. We denote henceforth by $T_{\text {b}}$ and $V_{\text {b}}$ the normal stress and normal velocity externally applied at $x=L$ whereas $c_{\text {ext}}$ is the external oxygen concentration. Two sets of simulation tests are performed. In the first set of simulations we set $T_{\text {b}} = V_{\text {b}}=0$ with the aim of investigating a static culture environment (see [57, 58]). In the second set of simulations we set $T_{\text {b}} = 100 {\mathrm {mPa}}$ and $V_{\text {b}} = 50 {\mathrm {\mu m s^{-1}}}$ as in [14]. These values are characteristic of a culture in a perfusion bioreactor where an external hydrodynamic shear stress is applied [55, 58, 63].

The first investigated question is the effect of the input model parameter amount *A* of cell density at the beginning of the culture process ($t=0$) and at the pore wall ($x=0$). For cells and ECM we set $\phi _\eta (x,0) = A_\eta \exp (-x/L_d)$, $\eta = {\text {n}}, {\text {v}}, {\text {q}}, {\text {ECM}}$, with $L_d = L/5$, and for each set of simulations we use the following values of *A*:

(IC1): $$A_n=0.005$$, $$A_\eta =0.001 \qquad \eta ={\text {v}},{\text {q}},{\text {ECM}}$$;
(IC2): $$A_n=0.05$$, $$A_\eta =0.01 \qquad \quad \eta ={\text {v}},{\text {q}},{\text {ECM}}$$.

The above values of $A_n$ and $A_{\eta }$ agree with the biophysical evidence that at the beginning of the growth process, proliferating cells are present in larger amount than the other cellular populations.

The second investigated question is the effect of the input model parameter cellular growth rate $k_{\text {g}}$. In our computations we use two values of this parameter, $k_{\text {g1}}$ and $k_{\text {g2}}$ (cf. Table 1). These two values are selected by comparison with the maximum specific cell growth rate $k_{\text {g0}}$ used in [63] in such a way that $k_{\text {g}} < k_{\text {g0}}$ corresponds to “low growth regime” whereas $k_{\text {g}} > k_{\text {g0}}$ corresponds to “high growth regime”.

The third investigated question is the effect of the input model parameter maximum value of the external oxygen concentration $c_{\text {ext}}$ supplied to the growing structure by the surrounding environment. To determine the effect of oxygen availability on biomass growth we set $c_{\text {ext}}(t) = c_{\text {sat}}$ and $c_{\text {ext}}(t) = c_{\text {thr}}$ for all $t \in [0, T_{end}]$, $c_{\text {sat}}$ and $c_{\text {thr}}$ being the saturation and threshold oxygen concentration, respectively (see Table 1).

For a synthetic representation of the isotropy indicator *r*, we define the following (equivalent) parameter $\xi = \xi (r)$ as $\xi (r) = 1$ if $r \le \bar{r}$ and $\xi (r) =0$ if $r>\bar{r}$. In the remainder of the discussion, no plot is reported for the fluid volume fraction $\phi _{{\text {fl}}}$ because this variable can be computed by post-processing using (8f). Simulations are run over the time interval $[0, T_{end}]$, with $T_{end}=30$ days, and the one-dimensional plots show the time evolution of the solid and fluid mixture components at the spatial coordinate $x=L/2$. The values of model parameters used in the numerical experiments are reported in Table 1.

## Discussion of simulation results

In this section we address a critical discussion of the more significant outcomes of the simulations of the model illustrated in Sect. 8. The adoption of the 1D setting has three points of strength. The first point of strength is that the simplicity of the geometry permits a verification of reliability of model predictions based on biophysical intuition. The second point of strength is that, despite being simple, the 1D setting preserves the main features of the 3D biomass growth process, permitting a comparison with experimental measurements. The third point of strength is that it is relatively easy to single out the presence of critical parameters in the mathematical formulation and investigate their quantitative influence on the evolution of mixture components.

**Fig. 9:**
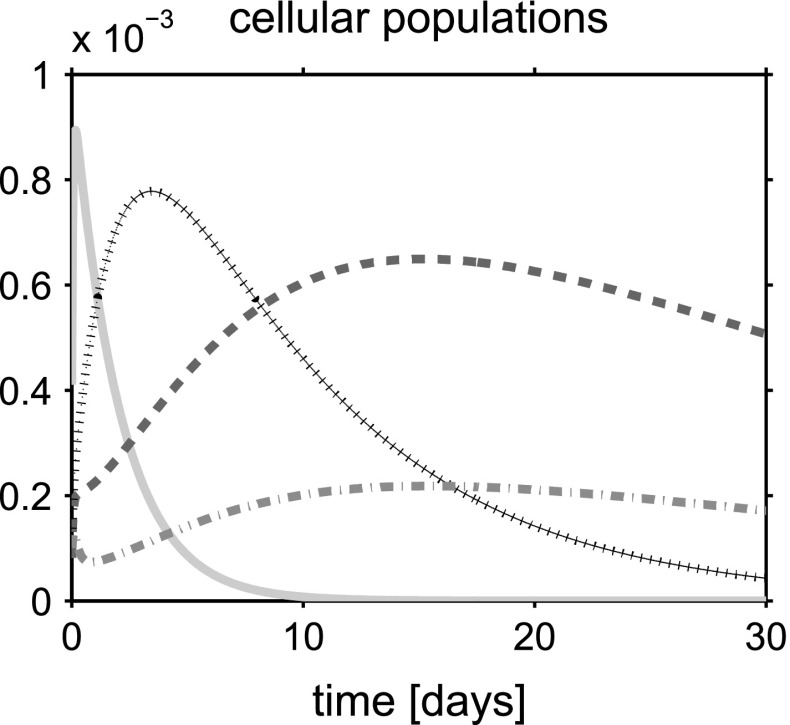
Temporal evolution of cellular populations and ECM in the static culture for $c_{\text {ext}}=c_{\text {sat}}$. Initial condition IC1; $k_{\text {g}} = k_{\text {g1}}$. *Solid line*: $\phi _{\text {n}}$; *dashed line*: $\phi _{\text {v}}$; dotted line: $\phi _{\text {q}}$; *dash-dot line*: $\phi _{\text {ECM}}$

**Fig. 10:**
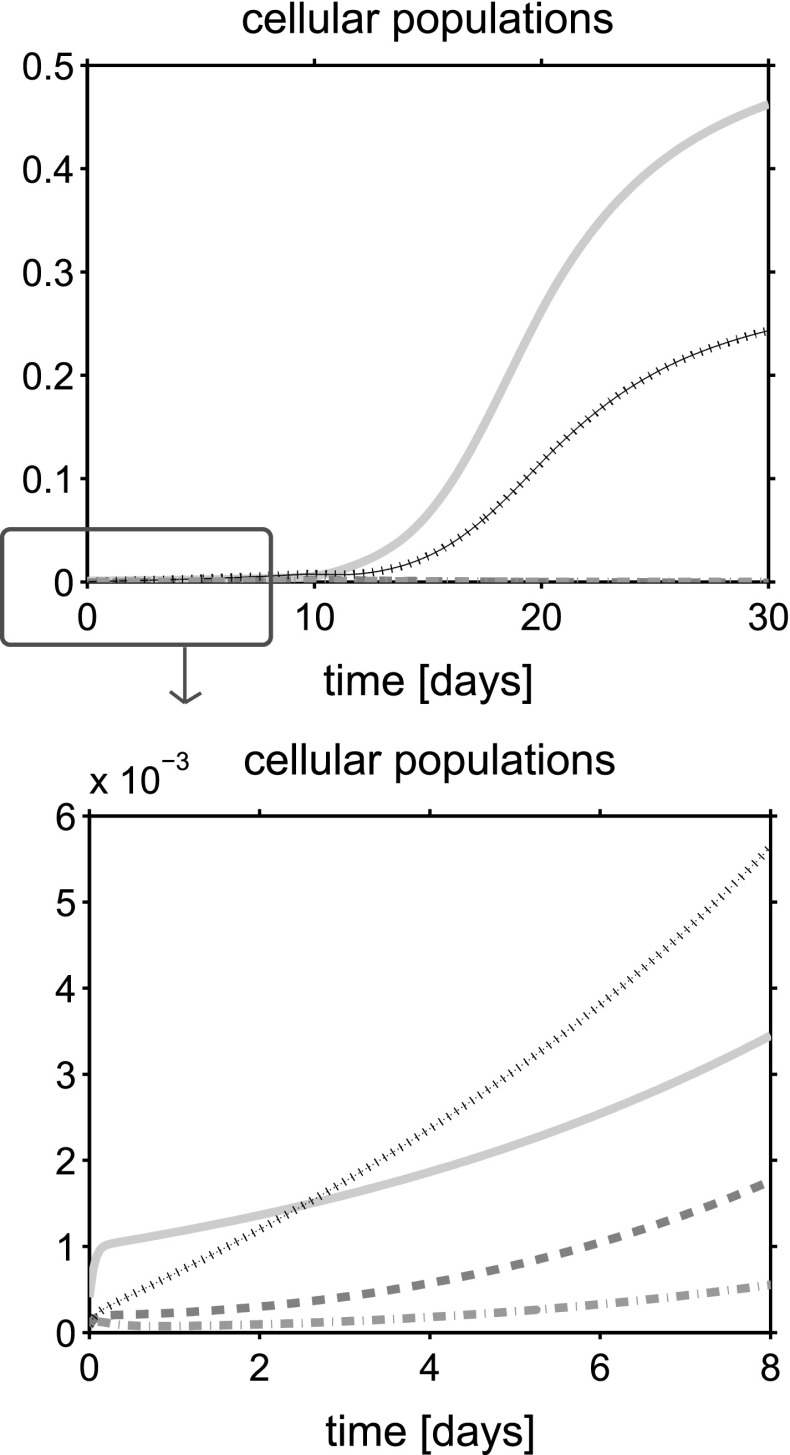
Temporal evolution of cellular populations and ECM in the static culture for $c_{\text {ext}}=c_{\text {sat}}$. Initial condition IC1. *Top*: $k_{\text {g}} = k_{\text {g2}}$. *Bottom*: $k_{\text {g}} = k_{\text {g2}}$, zoom of the first 8 days of culture. *Solid line*: $\phi _{\text {n}}$; *dashed line*: $\phi _{\text {v}}$; *dotted line*: $\phi _{\text {q}}$; *dash-dot line*: $\phi _{\text {ECM}}$

**Fig. 11:**
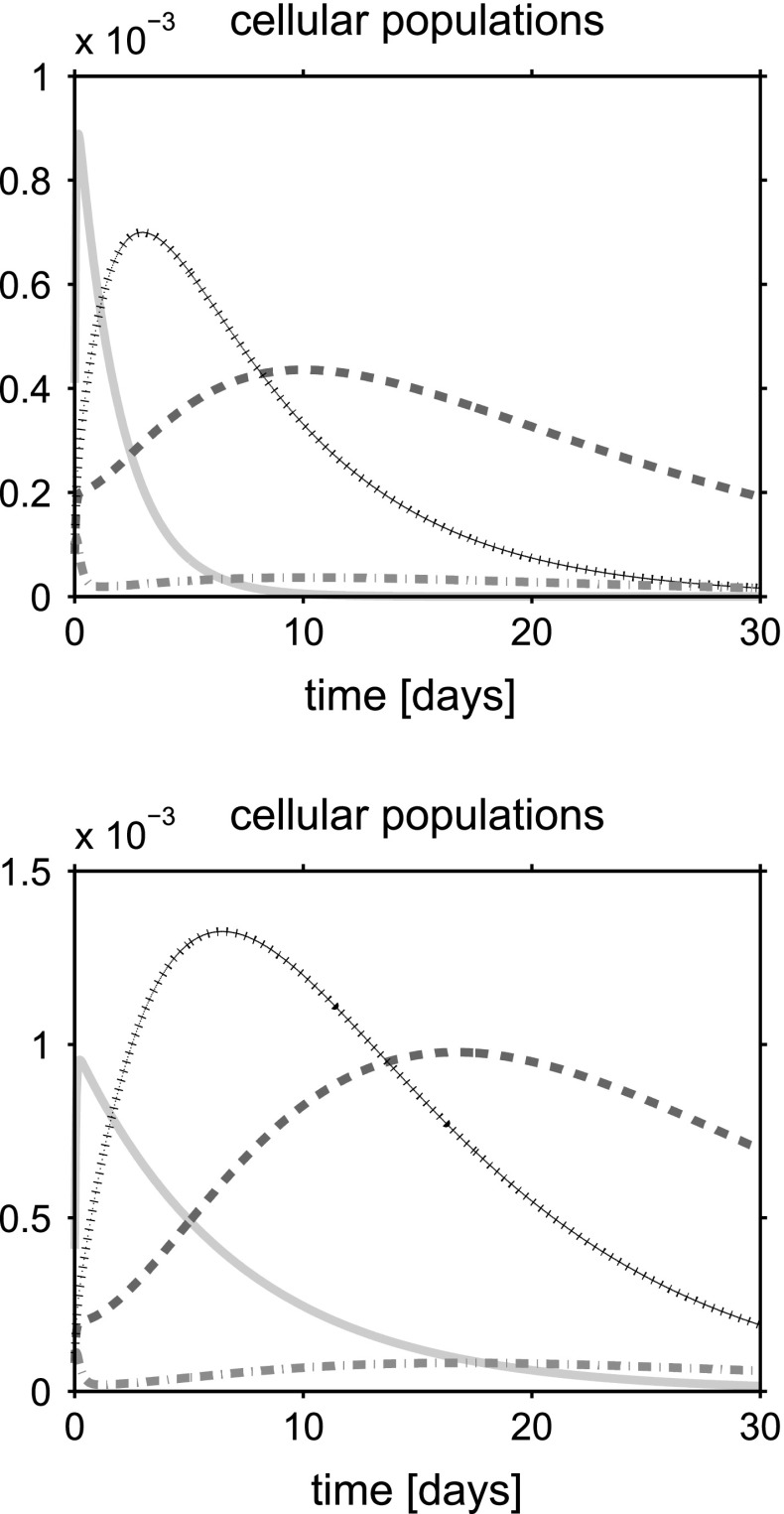
Temporal evolution of cellular populations and ECM in the static culture for $c_{\text {ext}}=c_{\text {thr}}$. Initial condition IC1. *Top*: $k_{\text {g}} = k_{\text {g1}}$. *Bottom*: $k_{\text {g}} = k_{\text {g2}}$. *Solid line*: $\phi _{\text {n}}$; *dashed line*: $\phi _{\text {v}}$; *dotted line*: $\phi _{\text {q}}$; *dash-dot line*: $\phi _{\text {ECM}}$

**Fig. 12:**
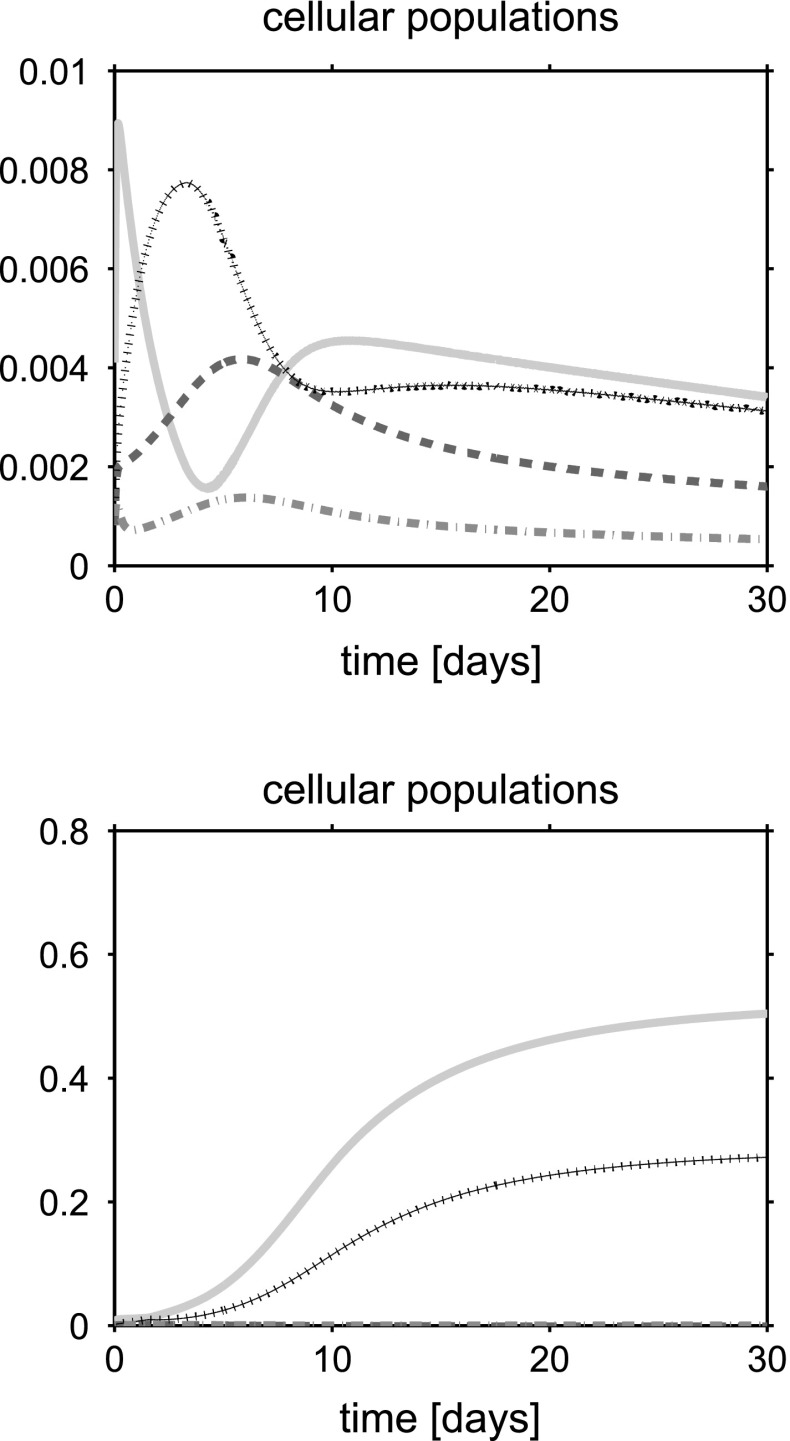
Temporal evolution of cellular populations and ECM in the static culture for $c_{\text {ext}}=c_{\text {sat}}$. Initial condition IC2. *Top*: $k_{\text {g}} = k_{\text {g1}}$. *Bottom*: $k_{\text {g}} = k_{\text {g2}}$. *Solid line*: $\phi _{\text {n}}$; *dashed line*: $\phi _{\text {v}}$; *dotted line*: $\phi _{\text {q}}$; *dash-dot line*: $\phi _{\text {ECM}}$

**Fig. 13:**
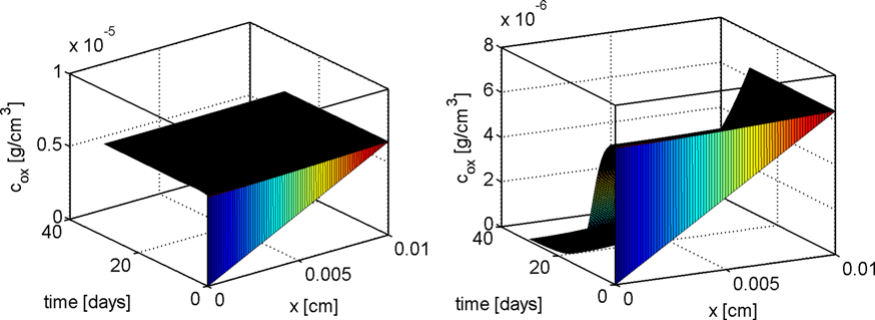
Spatial and temporal evolution of oxygen concentration in the static culture for $c_{\text {ext}}=c_{\text {sat}}$. Initial condition IC2. *Left*: $k_{\text {g}} = k_{\text {g1}}$. *Right*: $k_{\text {g}} = k_{\text {g2}}$. In the case of initial condition IC1 we observe a similar behavior of $c_{\text {ox}}$ except for a delayed decay

**Fig. 14:**
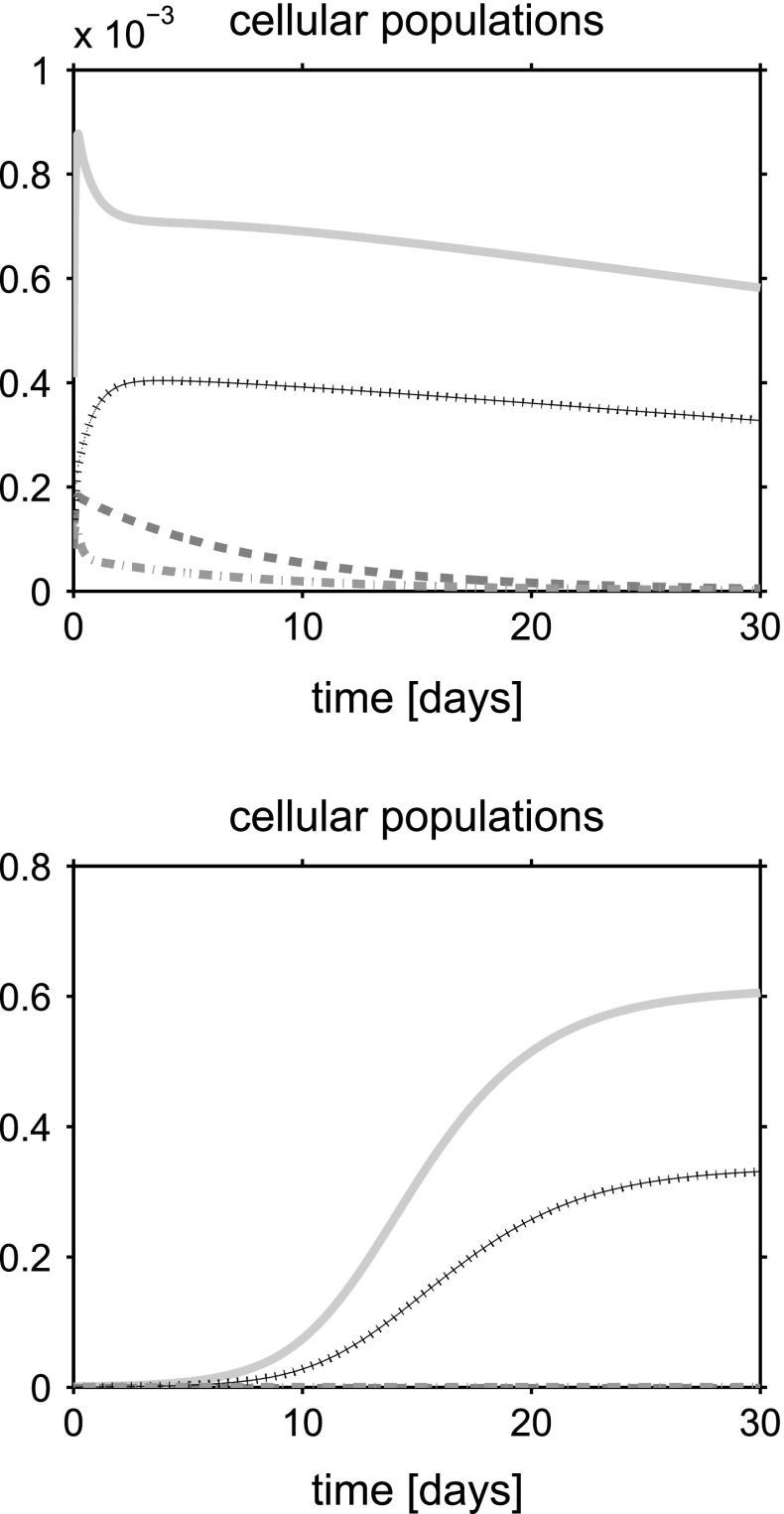
Temporal evolution of cellular populations and ECM in the perfused culture for $c_{\text {ext}}=c_{\text {sat}}$. Initial condition IC1. *Top*: $k_{\text {g}} = k_{\text {g1}}$. *Bottom*: $k_{\text {g}} = k_{\text {g2}}$. *Solid line*: $\phi _{\text {n}}$; *dashed line*: $\phi _{\text {v}}$; *dotted line*: $\phi _{\text {q}}$; *dash-dot line*: $\phi _{\text {ECM}}$

**Fig. 15:**
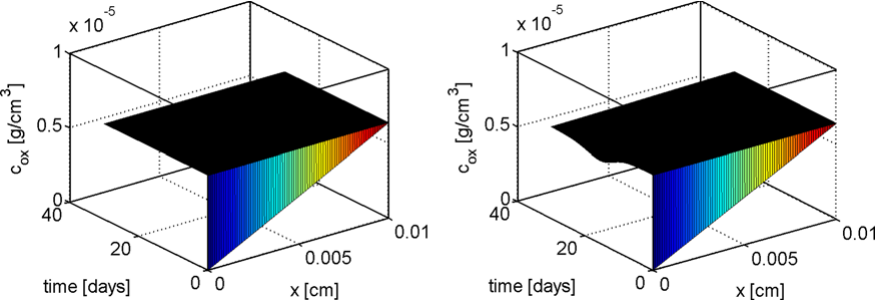
Spatial and temporal evolution of oxygen concentration in the perfused culture for $c_{\text {ext}}=c_{\text {sat}}$. Initial condition IC1. *Left*: $k_{\text {g}} = k_{\text {g1}}$. *Right*: $k_{\text {g}} = k_{\text {g2}}$

**Fig. 16:**
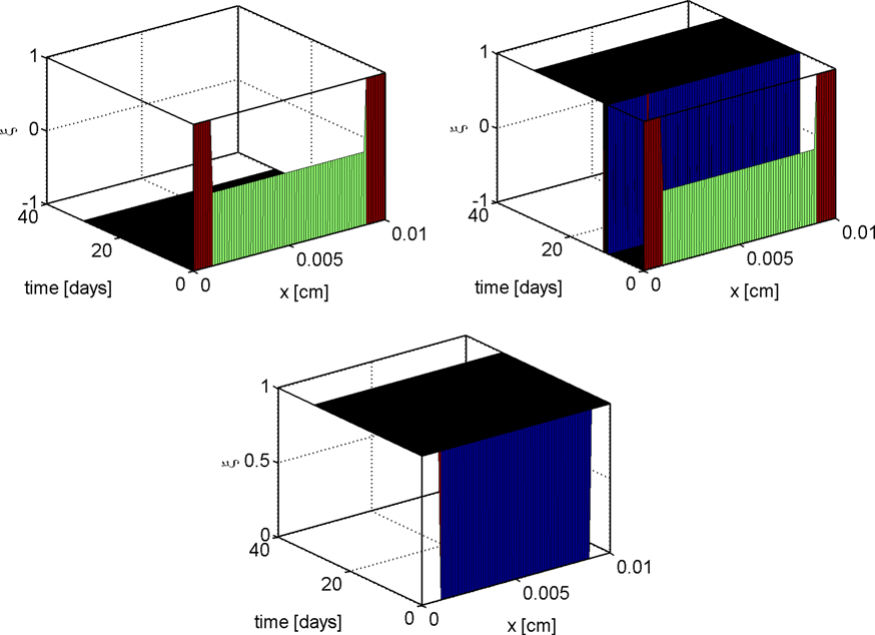
Spatial and temporal evolution of parameter $\xi$ in the static culture (*top*) and in the perfused culture (*bottom*) for $c_{\text {ext}}=c_{\text {sat}}$. Initial condition IC1. *Top left*: $k_{\text {g}} = k_{\text {g1}}$. *Top Right*: $k_{\text {g}} = k_{\text {g2}}$. *Bottom*: $k_{\text {g}} = k_{\text {g1}},k_{\text {g2}}$

**Fig. 17:**
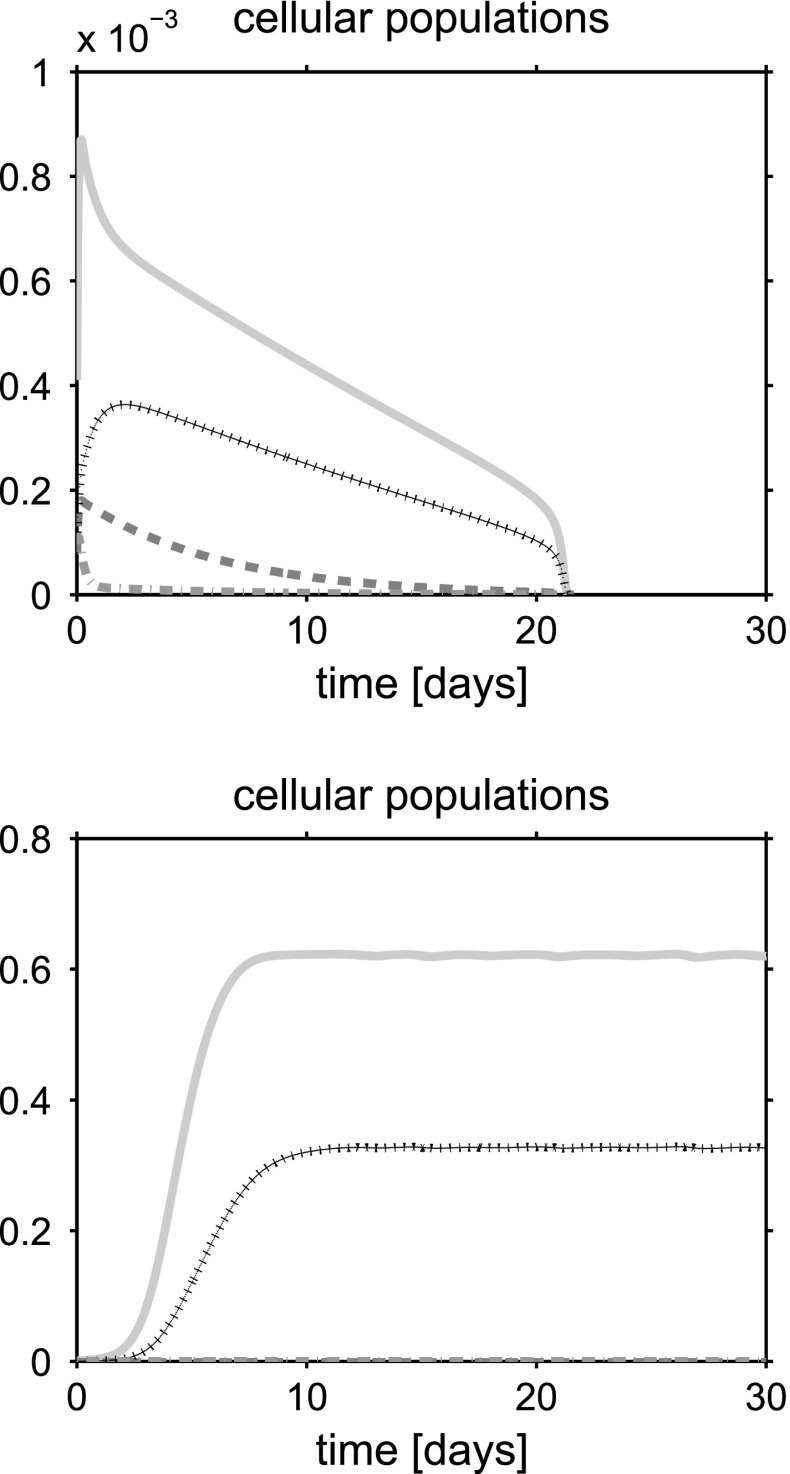
Temporal evolution of cellular populations and ECM in the perfused culture for $c_{\text {ext}}=c_{\text {thr}}$. Initial condition IC1. *Top*: $k_{\text {g}} = k_{\text {g1}}$. *Bottom*: $k_{\text {g}} = k_{\text {g2}}$. *Solid line*: $\phi _{\text {n}}$; *dashed line*: $\phi _{\text {v}}$; *dotted line*: $\phi _{\text {q}}$; *dash-dot line*: $\phi _{\text {ECM}}$

1. The illustrated numerical results indicate that the in vitro cell cultivation process is strongly sensitive to variations of (1) the initial seeding density of cells, (2) the value of the maximum growth rate and (3) the mechanical boundary conditions. In particular, the amount of seeded cells turns out to be determinant for cell responsiveness: initial cell density should be high enough to ensure optimal conditions for proliferation, but not so high that grow factors are rapidly depleted from the medium and the contact inhibition phenomenon prevents the formation of new colonies (see Fig. 13). To furtherly support this conclusion, Fig. 12 shows that at high seeding density no significant change in cell number is predicted by the model in the first stage of the culture because proliferating cells fluctuate around a mean value, that represents the average level of proliferation measured in the first 2 weeks of the experiments (see Fig. 3c of [11]). Furthermore the value of parameter $k_{\text {g}}$ influences the long-term behavior of the biomass. Model simulations indicate that if $k_{\text {g}}$ is smaller than the reference value $k_{\text {g,0}}$, cell metabolic activity and ECM synthesis significantly decrease in the cultured construct and are completely exhausted at about 10 days of culture (Figs. 9, 11 top). On the contrary, model predictions show that if the maximum growth rate exceeds the reference value, cell and ECM volumetric fractions increase until convergence to a finite value that represents a stable steady state of the mathematical system (Figs. 10, 11 bottom). This finding represents a favorable result from the experimental point of view, because it predicts the formation, at the end of the cultivation and under specific conditions, of the bio-artificial texture to be used for replacing damaged tissues, that constitutes the real aim of TE. A similar objective can be reached by conveniently assigning the mechanical boundary conditions at the interface between the biomass construct and the interstitial fluid. Model results indicate that when the biomass is stimulated by both external fluid velocity and pressure, even if $k_{\text {g}}$ is tuned on a under-threshold value, the amount of cells and ECM in the construct remains considerable until the end of the simulation (see Fig. 14 top panel). Such a behavior is mainly due to the fact that the external force exerted by the fluid gives rise to an anisotropic stress state that instantaneously propagates throughout the domain, as shown in Fig. 16 bottom, maintaining the anisotropic mechanical configuration in both low and high growth regimes. This mechanical stimulus is the sole responsible of the strong mitotic functional activity occurring within the biomass because, as evidenced in Fig. 15, oxygen consumption is practically absent. This outcome reinforces the notion that mechanical stimulation in perfused cultures may promote chondrogenesis and ECM production [17, 57, 58]. Actually, in order to achieve this optimal result, nutrient concentration at the fluid-biomass interface should not fall under a critical level otherwise cell functionality could be rapidly reduced until cell apoptosis (see Fig. 17 top). In these conditions, cell survival is ensured only if the growth rate $k_{\text {g}}$ is large enough (Fig. 17 bottom).
2. The behavior of the solid mixture components is in excellent agreement with the experimental trends obtained by cultivation of engineered tissues in bioreactors. In particular, the temporal evolution of construct cellularity and ECM content, especially for an under-threshold value of $k_{\text {g}}$, agree with experimental results shown in several papers [17, 23, 48, 72].
3. The characterization of the (an)isotropicity of the biomass intrinsic stress state through the equivalent parameter $\xi$ demonstrated to be a successful strategy to model the mechanical regulation of culture progression and to link the mechanisms occurring at the micro-scale level to the macroscopic functioning of the growing tissue (see [43]). Model predictions indicate that the parameter $\xi$ is an effective indicator of the propagation of the isotropic and anisotropic waves within the construct and allows an easy and immediate identification of the adhesion mechanisms developing at the single cell-level, that, accordingly, drive the evolution of the volumetric fraction $\phi _{\text {v}}$ and $\phi _{\text {n}}$ respectively.

## Conclusions and future perspectives

In the present article we propose a novel mathematical formulation based on the continuum assumption to describe the biomechanical sensitivity of articular chondrocytes. The natural application of our model is Tissue Engineering, a continuously growing discipline within the wider area of Regenerative Medicine, in which the control of cell response to multi-factorial stimuli is of utmost importance to obtain products suitable to clinical practice. However, it is worth noting that the proposed scheme may be used as well to describe more general settings in Cellular Biology, for example, the expansion of staminal cells.

The principal novelty of our contribution is the development of a model based on the use of Partial Differential Equations (PDEs) that incorporates the concept of “force isotropy” on the cell within the general and well established framework of poroelastic theory of mixtures and of cell population models. Specifically, the model translates into a simplified mathematical formalism, based on the use of suitably parametrized Heaviside functions, how the induced cytoskeletal tensional states trigger signalling transduction cascades regulating functional cell behavior, for example, the traslocation of specific transcription factors in the nucleus. According to the concept of force isotropy, it turns out that if cell adhesion-mediated traction forces have approximately the same strength over the cell surface, then the cell nucleus tends to maintain a roundish morphology, otherwise the cell nucleus tends to elongate. In the first case, the cell tensile condition is defined as “isotropic cytoskeletal tension” whereas in the second case the cell tensile condition is defined as “anisotropic cytoskeletal tension”.

Having defined the cytoskeletal stress characterization at the single cellular level, the next step of our approach is to build upon the concept of continuum-based approach to extend the above described description to the local stress tensor associated with the biological construct to mathematically represent the isotropic or anisotropic cell adhesion state. To this purpose, we generalize in a natural manner the previous definitions prescribing that if the anisotropic part of the local stress tensor is lower than a fixed threshold then the local stress state of the system is isotropic otherwise the local stress state of the system is anisotropic.

The final step of our model construction is to incorporate the above illustrated mechanobiological scheme within the setting of the theory of poroelasticity of a mixture composed by a solid and a multi-component fluid phases. The mixture represents the cellular construct in which several different cellular populations are well-mixed and oxygen delivery and consumption is taken into account to regulate in a dynamical manner the progressive fate of the evolving (macroscopic) tissue. The overall mathematical formulation consists of a system of conservation laws (mass and linear momentum) for the phases and components of the mixture that includes stress state and oxygen tension as main determinants of cellular culture evolution.

A thorough investigation of the PDE system is critically performed in a simplified 1D setting to allow an easy preliminary validation of the formulation. Extensive simulation tests outline a generally sound response of the computational model with respect to biophysical conjectures. In particular, numerical results indicate that the in vitro cell cultivation process is strongly sensitive to variations of (1) the initial seeding density of cells, (2) the value of the maximum growth rate and (3) the mechanical boundary conditions.

Below, we mention several future steps that we intend to take in the prosecution of this promising research activity.

1. A stability analysis of the homogeneous steady states of the dynamical system that describes the conservation of mass of the solid mixture components. Such a study will allow us to characterize the admissible range of values of model constitutive parameters that ensures the biophysical consistency of the proposed mathematical representation, in the same spirit as in [41] and [37].
2. The introduction of a visco-elastic component in the constitutive law for the total stress (as recently done in [7]). This extension of the model will allow us to perform a validation of the model and of the computational tool against available analytical solution and data (see [69]).
3. The inclusion of other mixture constituents, such as proteoglycan and collagen as done in [33]. This extension of the model will allow us to provide a more realistic biomechanical description of the growing tissue.
4. The extension of the computational algorithms to treat a fully three-dimensional representation of the scaffold pore to allow a deeper model validation against previous existing simulation results and experimental data (see, e.g., [19, 34, 56]). In particular, a 3D implementation of the model could provide a very interesting *in silico* scheme to simulate different levels of isotropy/anisotropy that otherwise should be reproduced *in vitro* at the price of complex engineering strategies, as described in [34]. Once a wide range of simulated mechanical configurations is available, it should be easier to relate cell response to the external mechanical stimuli and, consequently, to predict cell behavior in terms of cell adhesion, proliferation and differentiation. The 3D model could also be used to reproduce one pore of the microscaffold structures recently developed in [44] to mimic the native cellular environment. Cells confined into micropores are subject to similar environmental cues as *in vivo*, so that the behavior predicted by 3D computations could be directly compared with the *in vivo* cellular processes. Of course, passing to a 1D implementation to a fully 3D simulation tool requires to face and solve, at least, four computational challenges. The first challenge is the need of a flexible tool for the generation of an accurate geometrical description of the structure to be simulated. The preferable choice is to use tetrahedral elements and to this purpose a very good 3D mesh generator is the *open-source* program gmesh. The second challenge is the selection of a stable and accurate time-advancing discretization method. The obvious choice is to continue to emply the Backward Euler scheme. If a more accurate method is in order, the choice might fall on the second-order Trapezoidal or TR-BDF2 methods (for description and analysis, see [52], Chapter 11). The third challenge is the need of extending the selection of the stable and accurate finite element spaces used to approximate the various subproblems to be solved with the fixed-point map illustrated in Sect. 9.1. To this purpose, the Mini element and the Taylor-Hood pair are the best options for an accurate and stable treatment of the poroelastic equations, whereas the Edge Averaged Finite Element scheme investigated in [75] is a very effective method for accurately dealing with sharp layers in the nutrient concentration and/or cellular population profiles while ensuring the positivity of the computed solution. In the perspective of implementing the mechanobiological model within a 3D finite element framework a possible interesting programme might be to exploit the facilities of the software MP-FEMOS (Multi-Physics Finite Element Modeling Oriented Simulator) that has been developed by one of the authors [1, 39, 40, 61].

**Table 1:** Numerical values of model parameters used in the simulation tests

Symbol	Definition	Value	Units	References
$c_0$	$O_2$ concentration for $t=0$	$5\times 10^{-6}$	${\mathrm {g \; cm^{-3}}}$	This work
$c_{\text {sat}}$	$O_2$ saturation concentration	$6.4\times 10^{-6}$	${\mathrm {g\;cm^{-3}}}$	[14]
$c_{\text {thr}}$	$O_2$ threshold concentration	$1.6\times 10^{-6}$	${\mathrm {g\; cm^{-3}}}$	[38]
$c_{\text {apo}}$	$O_2$ apoptosis concentration	$3.2\times 10^{-7}$	${\mathrm {g\; cm^{-3}}}$	[38]
$K_{\mathrm {eq}}$	$O_2$ local mass equilibrium coefficient	0.1	-	[13]
$D_{{\mathrm {c}},{\text {s}}}$	$O_2$ diffusivity in the solid phase	$0.75\times 10^{-5}$	${\mathrm {cm^2\; s^{-1}}}$	[13]
$D_{{\mathrm {c}},{\text {fl}}}$	$O_2$ diffusivity in the fluid phase	$1\times 10^{-5}$	${\mathrm {cm^2\; s^{-1}}}$	[13]
$V_b$	Inlet velocity of perfusion fluid	$50\times 10^{-4}$	${\mathrm {cm\; s^{-1}}}$	[14]
$T_b$	Stress due to perfusion fluid	100	${\mathrm {mPa}}$	[14]
$\mu _{\text {fl}}$	Fluid dynamic viscosity at $20^\circ$ C	$1.002 \cdot 10^{-2}$	${\mathrm {g \; cm^{-1}\;s^{-1}}}$	[29]
$R_{\text {n}}=R_{\text {v}}$	$O_2$ consumption rate for n/v-cells	$3.9\times 10^{-8}$	${\mathrm {g\; (cm^3\; s)^{-1}}}$	[63]
$R_{\text {q}}$	$O_2$ consumption rate for q-cells	$10^{-8}$	${\mathrm {g\; (cm^3\; s)^{-1}}}$	This work
$K_{1/2}$	$O_2$ half saturation constant	$3.2\times 10^{-6}$	${\mathrm {g\; cm^{-3}}}$	[63]
$\beta _{\mathrm{A} \rightarrow \mathrm{B}}$	Transition rate from state A to state B	$10^{-5}$	${\mathrm {s^{-1}}}$	This work
$k_{\text {apo}}$	Apoptosis transition rate	$3.858\times 10^{-7}$	${\mathrm {s^{-1}}}$	[63]
$k_{\text {qui}}$	Quiescence transition rate	$3.858\times 10^{-7}$	${\mathrm {s^{-1}}}$	This work
$k_{\text {deg}}$	ECM degradation rate	$7.7\times 10^{-7}$	${\mathrm {s^{-1}}}$	[71]
$k_{\text {g0}}$	Maximum specific cell growth rate	$5.8 \times 10^{-6}$	${\mathrm {s^{-1}}}$	[63]
$k_{\text {g1}}$	“low” specific cell growth rate	$1 \times 10^{-7}$	${\mathrm {s^{-1}}}$	This work
$k_{\text {g2}}$	“high” specific cell growth rate	$1 \times 10^{-5}$	${\mathrm {s^{-1}}}$	This work
*E*	Expansion coefficient	20		[14]
$k_{\text {GAG}}$	GAG synthesis rate	$8.61\times 10^{-11}$	${\mathrm {cm^6\;(cell\;s\;g)^{-1}}}$	[14]
$K_{\text {sat}}$	Monod saturation constant	$1.927\times 10^{-6}$	${\mathrm {g\;cm^{-3}}}$	[15]
$D_\eta$	Cells and ECM diffusion coefficient	$1\times 10^{-9}$	${\mathrm {cm^2\; s^{-1}}}$	This work
$\lambda _\eta$	Cells and ECM Lamé’s parameter	$5.1937\times 10^{3}$	${\mathrm {dyne\,cm^{-3}}}$	[64]
$\mu _\eta$	Cells and ECM Lamé’s parameter	$1.8248\times 10^{3}$	${\mathrm {dyne\,cm^{-3}}}$	[64]
$\phi _{{\text {ECM}},{\mathrm {max}}}$	Maximum ECM volume fraction	0.1		This work
$R_{\text {cell}}$	Cell radius	$5 \times 10^{-4}$	${\mathrm {cm}}$	This work
$V_{\text {cell}}$	Cell volume	$5.236\times 10^{-10}$	${\mathrm {cm^3}}$	This work
$\tau _{\text {m}}$	Mitotic characteristic time	172800	${\mathrm {s}}$	[65]
$K_{\text {ref}}$	Reference permeability	$1.67 \cdot 10^{-5}$	${\mathrm {cm^{3} s\; g^{-1}}}$	This work

## Abbreviations

TE: Tissue engineering
ECM: Extracellular matrix
ACC: Articular chondrocyte cell
PDE: Partial differential equation
BVP: Boundary value problem
IV-BVP: Initial value/boundary value problem
